# An appraisal of the current status of inhibition of glucose transporters as an emerging antineoplastic approach: Promising potential of new pan-GLUT inhibitors

**DOI:** 10.3389/fphar.2022.1035510

**Published:** 2022-11-01

**Authors:** Mithlesh Kumar Temre, Ajay Kumar, Sukh Mahendra Singh

**Affiliations:** ^1^ School of Biotechnology, Institute of Science, Banaras Hindu University, Varanasi, India; ^2^ Deparment of Zoology, Institute of Science, Banaras Hindu University, Varanasi, India

**Keywords:** GLUT, neoplastic cells, pan-GLUT inhibitors, glutor, tumor metabolism

## Abstract

Neoplastic cells displayed altered metabolism with accelerated glycolysis. Therefore, these cells need a mammoth supply of glucose for which they display an upregulated expression of various glucose transporters (GLUT). Thus, novel antineoplastic strategies focus on inhibiting GLUT to intersect the glycolytic lifeline of cancer cells. This review focuses on the current status of various GLUT inhibition scenarios. The GLUT inhibitors belong to both natural and synthetic small inhibitory molecules category. As neoplastic cells express multiple GLUT isoforms, it is necessary to use pan-GLUT inhibitors. Nevertheless, it is also necessary that such pan-GLUT inhibitors exert their action at a low concentration so that normal healthy cells are left unharmed and minimal injury is caused to the other vital organs and systems of the body. Moreover, approaches are also emerging from combining GLUT inhibitors with other chemotherapeutic agents to potentiate the antineoplastic action. A new pan-GLUT inhibitor named glutor, a piperazine-one derivative, has shown a potent antineoplastic action owing to its inhibitory action exerted at nanomolar concentrations. The review discusses the merits and limitations of the existing GLUT inhibitory approach with possible future outcomes.

## Introduction

Neoplastic cells display altered carbohydrate metabolism, which has emerged as one of the targetable hallmarks of cancer (Hanahan and Weinberg, 2011; Senga and Grose, 2021; Hanahan, 2022). One of the prominent features of the reprogrammed metabolism in cancer cells concerns the predominance of glycolysis irrespective of the availability of O_2_, a phenomenon designated as the “Warburg effect.” Accelerated glycolysis helps neoplastic cells rapidly produce ATP and precursors of the anabolism (Warburg et al., 1927; Warburg, 1956; Lopez-Lazaro, 2008; Cassim et al., 2020). Mathematical models of accelerated glycolysis suggest that it lets the neoplastic cells produce a much higher amount of ATP than that derived from the normal Krebs cycle (Gillies and Gatenby, 2007). To maintain the unhindered supply of glucose for fueling glycolysis, cancer cells overexpress several nutrient transporters on their cell surface, among which one of the most prominent ones are the glucose transporters (GLUTs) (Ancey et al., 2018; Szablewski, 2022). Thus, irrespective of their etiologies, most neoplastic cells overexpress GLUT1 and GLUT3 isoforms, which have high efficiency for glucose transport (Ancey et al., 2018; Suwabe et al., 2021). Consequently, several upcoming anticancer therapeutic strategies are focused on designing effective approaches to achieve inhibition of one or more GLUT isoforms to interfere with the glucose uptake of cancer cells. Given the background mentioned above, the following review of literature discusses the current status of knowledge concerning: 1) the importance of sugars in carbohydrate metabolism of neoplastic cells; 2) the biochemistry of the functioning of glucose transporters; and 3) Emerging approaches for therapeutic targeting of GLUTs.

### The necessity of glucose for neoplastic cells

**FIGURE 1:**
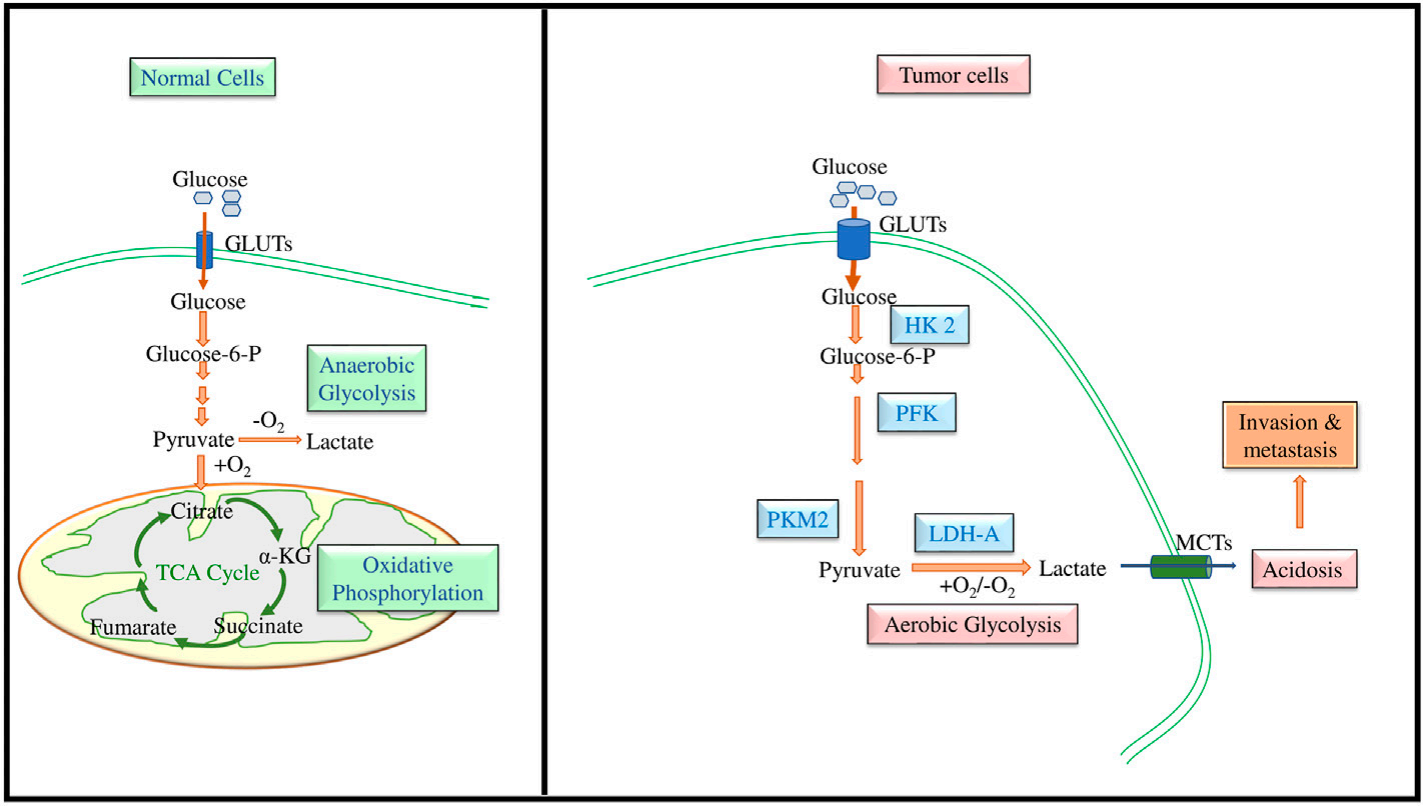
Role of GLUTs in the accelerated glycolysis of neoplastic cells. Neoplastic cells display an upregulated expression of GLUTs for increased glucose uptake, which is rapidly metabolized to lactate leading to the production of a high amount of ATP and biosynthetic precursors.

It has remained a hot and debated topic if sugars feed cancer. Among the diverse types of sugars, glucose is the simplest one, the most assimilable form of carbohydrates (Parker, 2020). Cancer cells metabolize glucose at a comparatively faster rate for which they need to have a high uptake of glucose. This feature of neoplastic cells was initially recognized by a well renowned German scientist Sir Otto Heinrich Warburg, Nobel laureate of 1931 in Physiology or Medicine, who noted that neoplastic cells consume a relatively higher amount of glucose (20 times more) than normal healthy counterparts (Warburg et al., 1927; Warburg, 1956). Further, Warburg also presented evidence demonstrating that cancer cells metabolized most of their consumed glucose *via* lactate fermentation instead of utilizing glucose metabolism through the TCA cycle. Figure 1 depicts the typical pathway of glucose metabolism in cancer cells in which glucose is metabolized to lactate compared to normal cells, which metabolize it through the TCA cycle. The role of glycolysis in neoplastic cells is accelerated on an average by 100 times (range: 20–300 times) (Vaupel and Multhoff, 2021). Though the glycolysis is inferior compared to the Krebs cycle for the generation of ATP, cancer cells still evolved to opt for glycolysis even in the presence of O_2_ owing to two possible reasons: 1) The rate of ATP production in cancer cells is approximately 100 times faster than normal cells and 2) glycolysis provides precursors for biosynthetic machinery (Lunt and Vander Heiden, 2011; Zhou et al., 2018). The metabolic switching of cancer cells is mainly attributed to HIF and its downstream signaling pathways (Semenza, 2010; Zhao T et al., 2014; Masoud and Li, 2015; Nagao et al., 2019; Lee et al., 2021).

### Glucose transporters (GLUTs)

Glucose uptake of cancer cells is facilitated by glucose transporters which belong to the solute carriers (SLC) family. The most prominent GLUTs are highly overexpressed in cancer cells and encoded by the SLC2A gene family (Thorens and Mueckler, 2010; Mueckler and Thorens, 2013; Navale and Paranjape, 2016; Galochkina et al., 2019; Echeverría et al., 2021). In addition, another group of membrane-associated glucose transporters belongs to the Na^+^/glucose cotransporters (SGLT) gene family designated as the SLC5A, which carries out the active transport of glucose (Wright et al., 2011).

**FIGURE 2:**
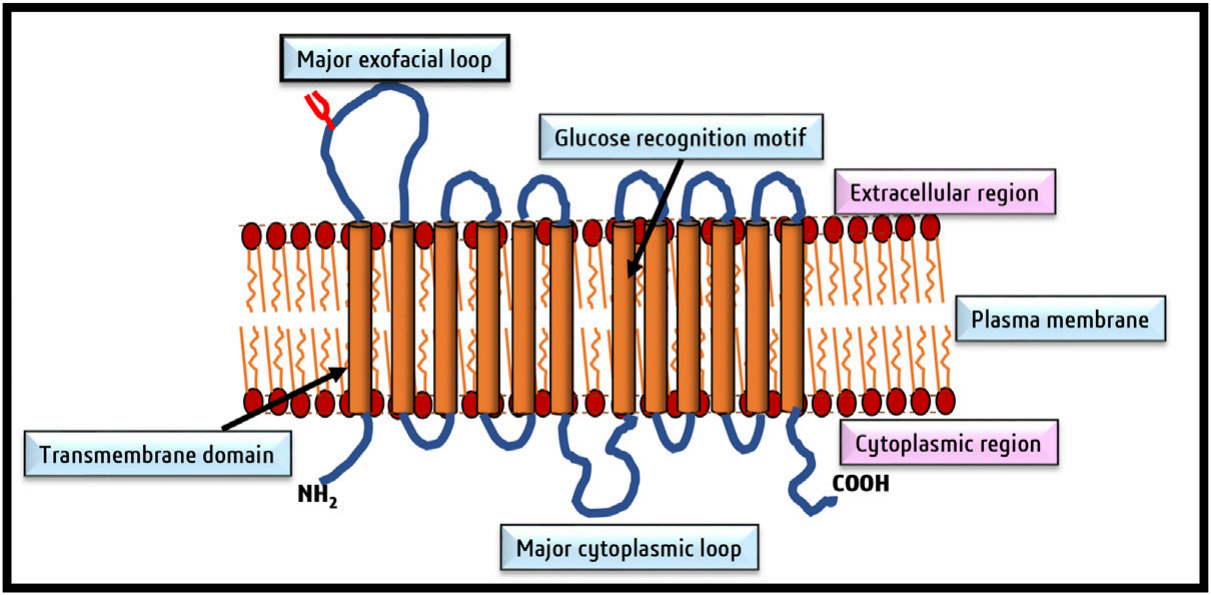
A typical topographical representation of GLUT (GLUT1). The figure shows the organization of the N and C termini, linker regions, and the 12 transmembrane domains of GLUT, which form an aqueous channel for glucose transport across the membrane.

The credit for discovering glucose transporters goes to LeFevre (1948), followed by advanced research to decipher their structure and functions primarily in the erythrocyte membranes (Widdas, 1952). Fourteen members now constitute the GLUT family with different affinities for glucose transport (Uldry and Thorens, 2004; Thorens and Mueckler, 2010; Mueckler and Thorens, 2013). Figure 2 depicts the typical structural organization of GLUT as downloaded from PDB Data Bank. A GLUT molecule is a glycoprotein with N-linked oligosaccharides composed of 500 amino acids arranged in an array of 12 transmembrane alpha-helices. Depending on the sequence differences, the nomenclature of the various members of the GLUT family was proposed (Joost and Thorens, 2001; Joost et al., 2002; Holman, 2020). According to the Heidelberg Unix sequence analysis, GLUTs are grouped into three classes: Class I, II, and III. Class I comprises GLUT1, GLUT2, GLUT3, GLUT4 and GLUT14. The class II members are GLUT5, GLUT7, GLUT9, and GLUT11. Class III constitutes GLUT 6, GLUT8, GLU10, GLUT12, and GLUT13/HMIT-1 (Ancey et al., 2018). The GLUT isoforms also display tissue-specific need-based variations in their expression pattern (Boado et al., 1994; Uldry and Thorens, 2004; Calvo et al., 2010). As neoplastic cells display essential dependency on glucose, they are reported to overexpress various isoforms of GLUTs (Table 1), particularly GLUT1, GLUT3, GLUT4, and GLUT12 (Martell et al., 1997; Szablewski, 2013; Barbosa and Martel, 2020; Pliszka and Szablewski, 2021). However, the expression of GLUT1 and GLUT3 is most ubiquitous in all cancers (Krzeslak et al., 2012; Barron et al., 2016); hence the following description of the literature review is focused on GLUT1 and GLUT3.

**TABLE 1:** Cancer genome-based representative distribution of GLUTs in various cancer cells.

Name of GLUT isoform	Representative neoplastic cells	Name of normal cells/tissues	References
GLUT1	Head and neck, brain, thyroid, pancreatic, breast, esophageal, gastric, renal, lung, cutaneous, adenocarcinoma, hepatocellular cholangiocellular carcinoma (HCCC), colorectal, bladder, endometrial, prostate, ovarian and cervical cancer, penile and hematological malignancies, sarcomas, laryngeal carcinomas	Erythrocytes, endothelial and epithelial cells, glandular cells, placenta, astrocytes, and cardiac muscles	Ancey et al. (2018); Barron et al. (2016); Carvalho et al. (2011); Ding et al. (2016); Gaber et al. (2021); Pliszka and Szablewski, (2021); Pragallapati and Manyam, (2019); Shim et al. (2013); Heydarzadeh et al. (2022); Zhao T et al. (2014)
GLUT2	Hepatocellular carcinomas, cholangiocellular carcinoma, breast, gastric cancers	Pancreatic islet cells	Godoy et al. (2006); Lin et al. (2016); Mueckler, (1994); Schmidl et al. (2021b, 2021a)
GLUT3	Non-small cell lung cancer, breast cancer, brain tumors, bladder, laryngeal, prostate, gastric, head and neck, ovarian, oral squamous cancer, astrocytoma, choriocarcinoma, retinoblastoma, rhabdomyosarcoma, pancreatic cancer, endometrial carcinoma	The brain, especially in neurons, testis, uterus, prostate, pancreas	Boado et al. (1994); Burant and Bell, (1992); Krzeslak et al. (2012); Meneses et al. (2008); Yamamoto et al. (1990)
GLUT4	Breast, lung, gastric, and pancreatic cancer	Insulin-sensitive tissue adipocytes and skeletal muscle	Adekola et al. (2012); Chadt and Al-Hasani, (2020); Richter and Hargreaves, (2013); Stuart et al. (2009); Torrance et al. (1997)
GLUT5	Liver carcinoma, lung cancer, renal cell carcinoma, breast and prostate cancer	Plasma membranes of small intestinal epithelial cells, kidney, testis, muscle	Chan et al. (2004); Godoy et al. (2006); Li et al. (2011); Phay et al. (2000); Yamamoto et al. (1990)
GLUT6	Several cancers, including endometrial cancer	Brain, spleen, and leukocytes	Godoy et al. (2006); Nualart et al. (2009); Pyla et al. (2013); Uldry and Thorens, (2004)
GLUT7	Not determined in cancer cells	Colon epithelium, prostate	Mueckler, (1994)
GLUT8	Multiple myeloma	Testis, adrenal gland, liver, spleen, lung	Maria et al. (2015); Shikhman et al. (2001)
GLUT9	Hepatocellular carcinoma, melanoma	Liver, kidney, lung, leukocytes, small intestine	Godoy et al. (2006); Phay et al. (2000)
GLUT10	Gastric cancer	Adipose tissue	Pyla et al. (2013)
GLUT11	Multiple myeloma	Several cell types, including heart, muscle, kidneys	McBrayer et al. (2012)
GLUT12	Breast and prostate cancer cells	Heart, small intestine, skeletal muscles	Bakht et al. (2020); Barron et al. (2012); Stuart et al. (2009); White et al. (2018)

The GLUT1 and GLUT3 display about 64% resemblance of amino acid composition. Deng and Yan (2016) have elegantly described the crystal structure-dependent details of GLUT1 and GLUT3. GLUT1 comprises 492 amino acids from aa 9–455 GLUT constitutes prominent canonical facilitator super family (MFS) protein folds (Deng et al., 2014; Galochkina et al., 2019; Drew et al., 2021). Twelve transmembrane α helices are linked with extracellular and cytoplasmic linker amino acids. The structural organization of GLUT1 and GLUT3 has been reported to exist in three conformational states.

**FIGURE 3:**
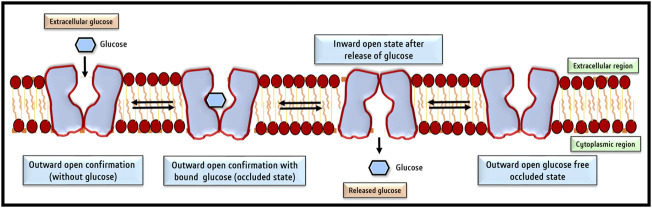
Conformational changes of GLUT facilitate glucose transport across the cell membrane. The four conformational changes oscillate between 1) Outside open conformation facilitating loading of glucose; 2) outward open conformation with bound glucose (occluded state); 3) inward open state following release of glucose and 4) shifting back to the outward open glucose-free state to capture of glucose. So basically, there are two main conformations the outside open (OOP) and inside open (IOP) states.

Despite amino acid differences, all GLUT isoforms possess a similar group structure, The central aqueous channel of GLUT1, through which glucose is passaged, comprises transmembrane domains 3,5,7, camp8 and 11 with amphipathic helices (Mueckler et al., 1985). Groups of Alvarez et al. (1987), Jung (1996), and Heinze et al. (2004) aided in the understanding of the structural organization of GLUT1 using specialized techniques like circular dichroism and Fourier transform infrared spectroscopy, mass spectroscopy, and scanning glycosylation mutagenesis (Alvarez et al., 1987; Jung, 1996; Heinze et al., 2004). Subsequently, the chemically predicted structures were reconfirmed using computer modeling techniques (Deng et al., 2014), followed by final corroboration of the predicted structures of GLUT1 by X-ray diffraction of crystal structures, with 12 transmembrane domains clustered into two units of six helical units (Deng et al., 2014; Holman, 2020). GLUT1 contains a single glucose binding site near the C terminal (Deng et al., 2015). Glutamine residues form the glucose binding site at 161, 282, 283, and 288 and tryptophan at 412 positions (Mueckler and Makepeace, 2009). The *in silico* structure and docking prediction tools have greatly added to deciphering the binding sites of glucose and inhibitors on GLUT and predicting the ‘Modus Operandi’ of the transport process (George Thompson et al., 2015). The two most acceptable models predict the transport of glucose by GLUT1. In the first model, it is predicted that glucose binding sites are located on two sides of the aqueous channel; one is located towards the exterior and the other towards the cytoplasmic side. Hence named as two site/flexed site transport model (Custódio et al., 2021). The glucose transport depends on a simple ligand exchange at these two binding sites. However, GLUT1 is demonstrated to transport glucose unidirectionally at a given time and hence is a uniporter. The second popular glucose transport model by GLUT1 is named the “alternating access model” (Lloyd et al., 2017). According to this model, GLUT1 undergoes transformational oscillations by alternatively opening on either side. Hence, GLUT1 is predicted to be an antiporter in this model (Chadt and Al-Hasani, 2020). The pioneering study of Deng et al. (2014) published in ‘Nature’ demonstrated the existence of four conformational states of GLUT1 based on its crystal structure. These four conformational states are designated as 1) outward open conformation with one substrate; 2) a ligand-bound state and occluded state; 3) an inward open state; and 4) a ligand-free and occluded state (Deng and Yan, 2016). A recent review by Galochkina et al. (2019) has summarized the current state of knowledge on the mechanism of glucose transport by GLUT1 by molecular dynamic simulation studies. According to the model proposed by this study, glucose transport by GLUT1 comprises three stages of a highly cooperative process. The movement of glucose involves its transition *via* a rotational movement with H-bonds (Galochkina et al., 2019), which is followed by the transformation of the outside open conformational state to the ‘inside open’ conformational state, through which the queued glucose molecule is released in the cytoplasm (Galochkina et al., 2019). The cytoplasmic glucose gets phosphorylated immediately by the action of hexokinase; hence, the concentration of free glucose in the cytoplasm remains at a very low concentration, which in turn becomes the driving force for the passive transport of glucose by GLUT (Medina and Owen, 2002). Figure 3 depicts various conformational changes which facilitate glucose transport across the cell membrane. This mode of glucose transport by GLUT is also designated as gated pore and rocker switch mechanism (Nomura et al., 2015), membrane phospholipids associated cell signaling has been reported to play a crucial role in regulating the signaling events responsible for the oscillation of GLUT conformational states (Hresko et al., 2016).

Nevertheless, in addition to glucose transport, GLUT1 also mediates the uptake of mannose, galactose, glucosamine, and ascorbic acid by cells (Uldry et al., 2002; Pliszka and Szablewski, 2021), which also work through the same operating system of altered conformational states (Pragallapati and Manyam, 2019). Moreover, the expression of GLUT on cell membrane displays clustered and focal distribution patterns, in which cell junctions and cytoskeletal elements play an essential role, as revealed by direct stochastic optical reconstitution microscopy (Yan et al., 2018). Almahmoud et al. (2019) performed a series of *in silico* studies to comprehend the mechanism of the aforementioned conformational changes in GLUT1. Their systemic molecular docking investigation revealed that the Phe291, Phe379, Gln380, Trp388, and Trp412 amino acid residues mediate the binding of glucose with GLUT1 and determine its conformational oscillation (Almahmoud et al., 2019). Further, the 12 transmembrane domains of GLUT1 are clustered in a replicated trimeric organization, as revealed by crystallographic studies (Holman, 2020). Crystallographic studies on GLUT1 have also revealed additional insight into the conformational states (Holman, 2020). Some amino acids are crucial in maintaining various conformational states, particularly the hydrophobic residues at 291, 292, 293, 294, 386, and 387 positions (Holman, 2020).

The expression of GLUTs in normal healthy cells and transformed cells is a highly regulated process, depending on the glucose needs of specific cell types (Navale and Paranjape, 2016). However, the precise mechanism of the gene expression and membrane transporters of GLUT1 and GLUT3 have still not been fully deciphered. Neoplastic cells largely depend on cAMP-dependent secondary messenger signaling for regulating the expression of GLUT1 and GLUT3 (Meneses et al., 2008). In addition, PI3K/Akt pathway has also been demonstrated to facilitate GLUT1 expression (Hoxhaj and Manning, 2020).

### Regulation of GLUT expression

A survey of literature strongly indicates the involvement of multiple signaling messengers in the regulation of GLUT expression in cancer cells in which the prominent ones include Ca^2+^ (Diaz-Ruiz et al., 2011; Echeverría et al., 2021), CAMK (White et al., 2018), PI3K/Akt (Hoxhaj and Manning, 2020), mTOR (Shin and Koo, 2021), HIF1-α, p53 (Monde, 2018; Schwartzenberg-Bar-Yoseph et al., 2004), Matrix metalloproteinases (MMP) (Ito et al., 2002), RAS, PKC (Lee et al., 2015), NF-κB (Zha et al., 2015), AMPK (Zambrano et al., 2019), Phosphate and Tensin Homolog (PTEN) (Ancey et al., 2018), and Thioredoxin-interacting protein (TxNIP) (Ancey et al., 2018). Many of these critical signaling and regulatory molecules are integrated into a specific loop to regulate the expression of various GLUT isoforms. Zhao and Zhang (2016) have elegantly described critical regulatory and canonical pathways regulating GLUT expression: GLUT-PI3K, GLUT-mTOR, GLUT-HIFs, GLUT-RAS, GLUT-MMP, and GLUT-p53 (Zhao and Zhang, 2016). Upstream to these cell-associated signaling mediators regulating GLUT expression, several extrinsic factors are also key players in modulating GLUT expression. Among these, proinflammatory cytokines have been reported to play a crucial role in regulating the expression of GLUT1 and GLUT3. GLUT regulatory cytokines include TNFα (Shikhman et al., 2001; Straus, 2013), IL-1β (Phillips et al., 2005), TGF-β (Andrianifahanana et al., 2016), IFN-y (Freemerman et al., 2014), IGF1 (Actis Dato et al., 2021), and IL-13 (Wieman et al., 2007). These cytokines indirectly regulate GLUT expression *via* their ability to modulate one or more signaling pathways. For example, IL-1β regulates GLUT expression by modulating PKC, PI3K, p38, and cJUN activation. Likewise, proinflammatory cytokines like IL-13 and IFN-y modulate GLUT1 expression *via* NF-κB activation (Freemerman et al., 2014; Tan et al., 2018). In addition to cytokines, several regulatory RNAs are also reported to regulate GLUT expression. Non-coding RNAs, including several miRNAs, have been demonstrated to regulate GLUT expression (Kong et al., 2016; Hu et al., 2019). Long non-coding RNA, HOTAIR, has been shown to regulate GLUT by its ability to modulate NFkB (Obaid et al., 2021). Extrinsic factors like iron availability also affect GLUT expression (Potashnik et al., 1995).

Importantly several hormones also play a crucial role in regulating GLUT expression. Gender-specific hormones like estrogen (Medina et al., 2004; Nualart et al., 2009), testosterone (Wilson et al., 2013; Mitsuhashi et al., 2016), and progesterone (Medina et al., 2003; Medina et al., 2004; Frolova et al., 2009; Nualart et al., 2009) whereas non gender-specific hormones like thyroid hormone (Torrance et al., 1997; Brunetto et al., 2012; Ding et al., 2016), insulin (Brosius et al., 1992; Stuart et al., 2009; Maria et al., 2015), and pituitary hormones (growth hormone, gonadotropin releasing hormone (GnRH), luteinizing hormone (LH), follicle stimulating hormone (FSH), and somatotropins) (Kilgour et al., 1995; Harris et al., 2012; De Los Santos, 2013; Kim and Park, 2017; Nicholas et al., 2020) have been implicated in the regulation of GLUT expression. However, like cytokines, the modulatory effect of hormones in GLUT expression is mediated *via* the involvement of one or more aforementioned signaling mediators and pathways (Kilgour et al., 1995; Shikhman et al., 2001; Phillips et al., 2005; Freemerman et al., 2014; Santos et al., 2014; Maria et al., 2015; Ding et al., 2016; Takaguri et al., 2016; Kim and Park, 2017). Nevertheless, the blend of cytokines, hormones, and specific signaling mediators is also likely to manifest cancer cell-specific differences in the expression of various GLUTs. Nevertheless, physical activities like exercise also modulate GLUT expression *via* CAMK and AMPK-associated signaling pathways (Röckl et al., 2007; Richter and Hargreaves, 2013).

**FIGURE 4:**
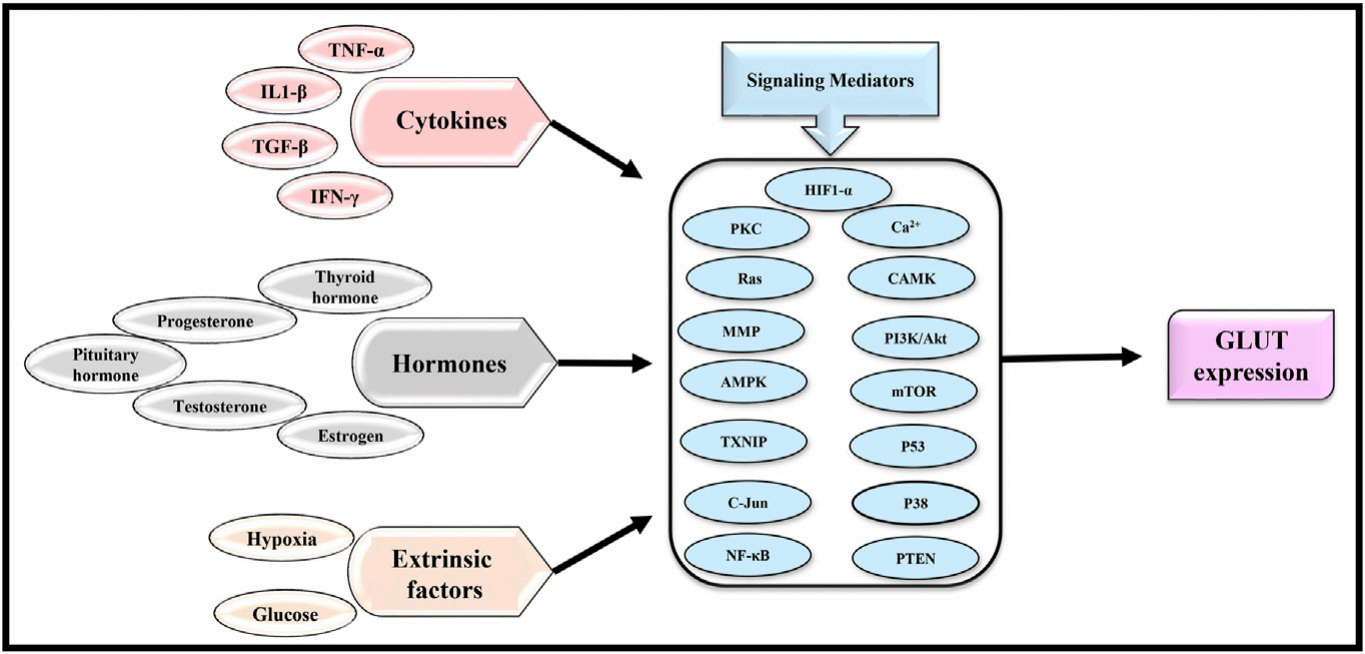
Regulation of GLUT expression in cancer cells. Indicated extrinsic and intrinsic factors can regulate the expression of GLUTs *via* the mediators of a plethora of signaling messengers, the blend of which could vary in a cancer-specific manner.

Further, the role of hypoxemic conditions and HIF1α in regulating GLUT expression needs a little more attention as HIF1α is the master regulator of tumor metabolism (Nagao et al., 2019). Experimental reports strongly indicate a correlation of HIF1α to the expression of various GLUT isoforms (Semenza, 2010; Mattmiller et al., 2011; Semenza, 2013; Sadlecki et al., 2014; Seleit et al., 2017; Kierans and Taylor, 2021). The modulation of GLUT expression by HIF1α is also mediated by the co-involvement of other signaling messengers like RAS (Moldogazieva et al., 2020; Ghanavat et al., 2021; Shin and Koo, 2021). Hypoxic conditions of the tumor microenvironment also regulate GLUT gene expression *via* modulation of HIF1α and its downstream signaling events (Roy et al., 2020; Li et al., 2021). Nevertheless, even glucose levels in the external milieu serve as essential triggers of GLUT1 and GLUT3 expression *via* HIF1α mediated regulation of GLUT gene expression (Hayashi et al., 2004; Yang et al., 2014; Zhao and Zhang, 2016; Seleit et al., 2017). Hypoxia and glucose levels have been reported to modulate mRNA levels of GLUT1, GLUT3, GLUT8, GLUT9, GLUT10, and GLUT12 (Frolova et al., 2009; Frolova and Moley, 2011; Kido et al., 2020), indicating the importance and central role of these extrinsic factors on pan-GLUT expression in which HIF1-α has a central role. Nevertheless, some evidence also indicates HIF1-α independent regulation of GLUT expression (Macheda et al., 2005). Such variations could be dependent on the etiology of various cancer cells. Figure 4 shows the primary regulators of GLUT expression and their downstream signaling mediators.

## Inhibition of GLUT as a promising anti-cancer approach

Considering the crucial role of glucose supply for sustaining accelerated tumor metabolism and most malignant cells’ overexpression of GLUT1 and GLUT3, strategies are being devised to effectively inhibit GLUTs in a neoplastic cell-specific manner. Nevertheless, as discussed earlier in this literature review, many cancer cells also overexpress other isoforms of GLUTs, SGLT1, and SGLT2 for sugar uptake (Szablewski, 2013; Wright, 2020; Pliszka and Szablewski, 2021; Szablewski, 2022). Thus, several approaches are envisaged for targeted inhibition of such transporters, particularly the GLUTs. Significantly, the effectiveness of any GLUT inhibition strategy will depend on the strength of the binding of a given inhibitor. Availability of the molecular structures of GLUT inhibitors and *in silico* tools has significantly helped to characterize GLUT inhibition.

Moreover, the availability of the crystal structure of GLUT1 and GLUT3 has further strengthened the approaches for exploring their effective inhibitors. In this context, searching and developing such inhibitors will be desirable, which can cause a pan-GLUT inhibition. This approach will help overcome the adaptability of neoplastic cells to recruit additional GLUTs for glucose uptake. Moreover, it is also essential to explore inhibitors that can inhibit GLUTs at minimal concentrations; this will aid in avoiding toxicity to normal cells with a low level of GLUT expression. In addition to the ongoing search for ideal small molecule inhibitors for GLUTs, other approaches mainly focused on inhibiting GLUT expression at the gene level are also under active investigation. These approaches include anti-GLUT antibodies, antisense DNA, shRNA, miRNAs, long non-coding (LNC), and Hox antisense intergenic RNA (HOTAIR) (Rastogi et al., 2007; Liu et al., 2018; Hu et al., 2019; Liu et al., 2021). Given the rapid progress in this field, the current status of GLUT inhibition strategies is discussed in the following section of the literature review.

### GLUT inhibitors of natural origin

Small molecule GLUT inhibitors mainly belong to natural, seminatural and synthetic chemical origins (Shriwas et al., 2020). have elegantly summarized the GLUT inhibitors of herbal origin. Vick et al. (1973) introduced the first herbal GLUT inhibitor named “Phlorizin,” which was followed by a series of discoveries of several other GLUT inhibitors of plant origin which belong to various chemical categories, including alkaloids, flavonoids, and other oxygen heterocyclic and phenolic compounds (Shriwas et al., 2020). Among these diverse categories of GLUT inhibitors of plant origin, those identified with antineoplastic action include more than 25 compounds, to name a few of them: apigenin (Gonzalez-Menendez et al., 2014), curcumin (Gunnink et al., 2016; Soni et al., 2021), genistein (Vera et al., 1996; Pérez et al., 2011), naringenin (Memariani et al., 2021), oridonin (Yao et al., 2017), phloretin (Wu et al., 2018; Wang et al., 2022), phlorizin (Vick et al., 1973; Kwon and Levine, 2007; Shriwas et al., 2020), quercetin (Schmidl et al., 2021a), resveratrol (Jung et al., 2013; Gwak et al., 2015; Zambrano et al., 2019; Samec et al., 2020), silybin (Zhan et al., 2011), and vinblastine (Shriwas et al., 2020). Although many of these GLUT inhibitors display a promising antineoplastic potential, the precise mechanism of the inhibitory action of most remains elusive, which needs to be deciphered for their optimal utilization in antineoplastic therapeutics.

### Cytochalasin B

A cell-permeable mycotoxin named cytochalasin B, which is also a microfilament poison and used to regulate cytoskeletal elements is yet another GLUT inhibitor of natural origin that is extensively investigated for its mechanism of GLUT inhibition and utility for antineoplastic applications (Jung and Rampal, 1977; Devés and Krupka, 1978; Klip and Pâquet, 1990; Granchi et al., 2016; Kapoor et al., 2016). Kapoor et al. (2016) published an elegant article regarding the mechanism of the GLUT-1 inhibitory action of cytochalasin B along with two phenylalanine amides. This study also showed that the GLUT-1 inhibitory action of cytochalasin B could be manifested in mM concentration ranges. The study utilized crystallography and *in silico* docking tools to decipher the mechanism of GLUT1 inhibition and identified the amino acids and binding sites through which cytochalasin B exerted its inhibitory action. However, being a blocker of microfilament polymerization, its utility for antineoplastic application *in vivo* needs to be verified vis à vis validation of safety for other organs and tissues. Moreover, the cytoskeleton plays a crucial role in the functioning of many tumor-infiltrating cells like macrophages and dendritic cells, where microfilament poisoning by cytochalasin B needs to be controlled while utilizing it for anticancer therapeutic approaches.

### Glucopiericidin A

Glucopiericidin A is yet another highly potent GLUT inhibitor of natural origin with an IC50 value of 22 nM compared to 500 nM for cytochalasin B (Kitagawa et al., 2010). The group of glucopiericidin A members are produced by *streptomyces* of the actinomycetes family. Glucopiericidin A has been remarkable for its low LD50 values and is predicted to have less toxicity (Zhou and Fenical, 2016). Though initially discovered as a filopodia protrusion inhibitor (Kitagawa et al., 2010), it was later discovered that glucopiericidin A could simultaneously inhibit both mitochondrial respiration (Hall et al., 1966) and glycolysis (Kitagawa et al., 2010) by its ability to inhibit GLUT leading to declined ATP production. Moreover, glucopiericidin A is also shown to interfere with tyrosine kinase-based signaling (Zhou and Fenical, 2016). It has been demonstrated that glucopiericidin A synergies with low glucose levels for inducing cell death in the pancreatic, lung, and other cancer cells (Palorini et al., 2013). However, the precise mechanism of GLUT inhibition by glucopiericidin A remains elusive. Moreover, its antineoplastic action needs to be assessed in a broader spectrum of targets, and its specificity on cancer cells remains undetermined. It remains elusive whether it can directly inhibit GLUT from interfering with glycolysis or *via* glucose phosphorylation. The GLUT inhibiting potential of glucopiericidin A solely depends on maiden experiments displaying its ability to inhibit the uptake of 2-deoxyglucose (2DG) (Imoto, 2019). Thus, the antineoplastic potential of glucopiericidin A needs further validation.

### Polyphenols

Polyphenols are a group of natural compounds found in many plants with reported antineoplastic potential (Keating and Martel, 2018). Polyphenols like apigenin, silibin, kaempferol, gossypol, naringenin, phloretin, genistein, resveratrol, herpertin, quercetin, myricetin, and catechin are reported to inhibit glucose uptake in several neoplastic cell lines, by their ability to inhibit GLUTs at both gene and protein expression level (Keating and Martel, 2018; Ji et al., 2019; Kang et al., 2019). On the other hand polyphenols like quercetin are reported to competitively inhibit GLUT1 (Hamilton et al., 2018; Salehi et al., 2020), whereas for some other polyphenols the mechanisms of GLUT inhibition remain unclear. Johnston et al. (2005) and Farrell et al. (2013) reported that dietary polyphenols decrease glucose uptake in the Caco-2 cell line, possibly depending on their ability to inhibit GLUT and SGLT1. Moreover, polyphenols like curcumin, genistein, and quercetin can improve the antineoplastic action of glycolytic inhibitors in cell lines of myeloid leukemia origin (de Blas et al., 2016). Moreover, phloretin, a polyphenol derived from apple, was shown to inhibit the proliferation of colorectal cancer cell lines by inhibiting GLUT2 and activating the p53-dependent signaling pathway (Lin et al., 2016). *In vitro* experiments on Caco-2 cells revealed that exposure to anthocyanin-rich plant extract inhibited the expression of SGLT1 and GLUT2 (Alzaid et al., 2013). However, for many polyphenols, the precise mechanism of their inhibitory action on GLUTs remains to be clarified. Among the most worked out polyphenols, apigenin and resveratrol are known to inhibit GLUT1 (Melstrom et al., 2008; Gwak et al., 2015), whereas phloretin, and quercetin are reported to inhibit GLUT1, and GLUT2 (Schmidl et al., 2021a) on the other hand silybin inhibits GLUT4 (Zhan et al., 2011). These studies on polyphenols also corroborate that eating fruits and a vegetable-rich diet abundant in polyphenols decreases cancer risk. It can be attributed mainly to the GLUT inhibitory potential of several dietary polyphenols. In addition, polyphenols manifest their anticancer action through other mechanisms, including modulation of cell signaling, cell survival, angiogenesis, immunopotentiation, hormonal regulation, and enzyme modulation (Niedzwiecki et al., 2016). The target cancers investigated for the anticancer action of polyphenols include prostate, colon, breast, lung, bladder, pancreas, spleen, and leukemia (Niedzwiecki et al., 2016; Montané et al., 2020).

### Small inhibitory molecules

**FIGURE 5:**
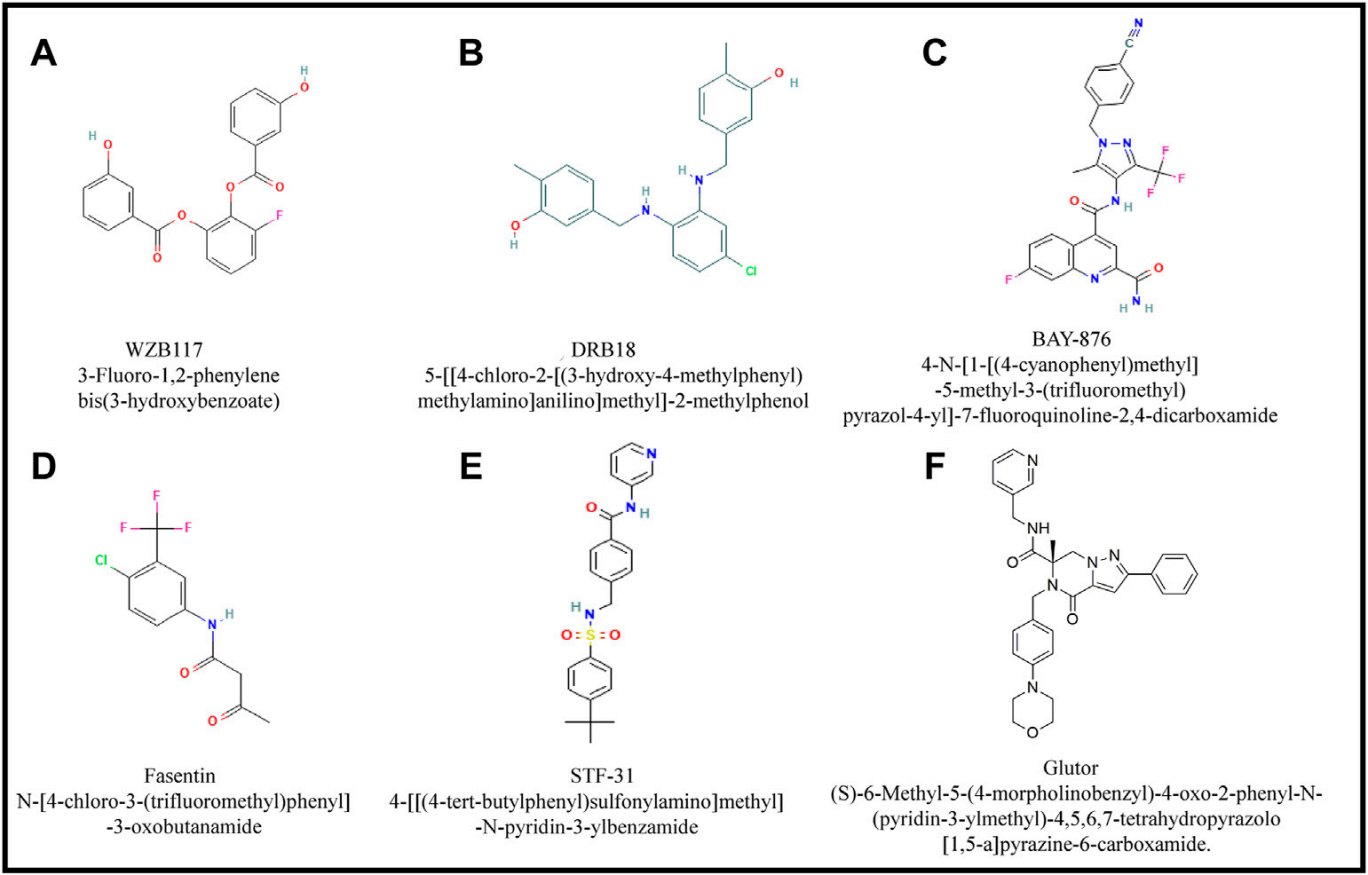
Major small inhibitory molecules of GLUTs. Figures **(A-F)** show structural details of small GLUT inhibitory molecules. Most of these molecules have overall common structural organization except few alterations in ring structure, constituent elements and their arrangement.

The main group of small inhibitory molecules of various GLUTs are principally aromatic compounds with unique imidazole, pyridine and pyrazole rings and most of them have a common overall structural organization with minor differences as depicted in Figures 5A–F.

### WZB 117

WZB 117 (Figure 5A) is a polyphenol-derived small molecule inhibitor of GLUT1 (Liu et al., 2012; Ojelabi et al., 2016; Chen et al., 2017). It exerts antineoplastic action with an IC50 value in the 10 µM concentration range on Hela, RKO, A549, and MCF7 cells of the cervix, endometrial carcinoma, lungs, and breast origin, respectively (Liu et al., 2012), accompanied by enhanced radio-sensitization (Zhao et al., 2016). Some mechanisms of the antineoplastic action of WZB117 have also been worked out, showing the implication of a declined GLUT1 expression along with a decrease in ATP levels and glycolytic enzymes (Liu et al., 2012). Moreover, WZB117 triggers an increase in the ATP sensing AMPK and a decline in cyclin E2 (Liu et al., 2012). The antineoplastic activity of WZB117 has also been evaluated *in vivo* (Liu et al., 2012). Interestingly WZB117 is also reported to inhibit cancer stem cells (Shibuya et al., 2014). However, one of the disadvantages of WZB117 is its instability owing to its ester bonds under aqueous physiological conditions, potentially the most significant limiting factor in its use in cancer therapeutic strategies. Moreover, it is described as inhibiting only GLUT1, which can be another limitation of its use for antineoplastic applications.

### DRB18

In a recent study, another promising GLUT inhibitor named DRB18 (Figure 5B), which belongs to a synthetic small inhibitory molecule, has been reported with the ability for GLUT inhibition as evaluated on A549 tumor-bearing nude mice and HER293 cell lines (Shriwas et al., 2021). One of the reported advantages of DRB18 is its relative stability compared to WZB117 (Roberts et al., 2020). So far, DRB18 has been shown to exert cytostatic action on around 60 cancer cell lines of diverse origins. The primary mechanism of the antineoplastic action of DRB18 was shown to be *via* increased endoplasmic reticulum oxidative stress (Shriwas et al., 2021). The pan-GLUT inhibitory ability of DRB18 was verified through in silico-based studies. The cytotoxic ability of DRB18 accompanied by inhibitory action on glucose uptake showed IC50 in the concentration range of 900 nM-9.0 µM (Shriwas et al., 2021). Moreover, DRB18 treatment caused a remarkable inhibition of glucose-dependent metabolic pathways like glycolysis, TCA cycle, purine, and pyrimidine synthesis (Shriwas et al., 2021). However, the GLUT inhibitory and antineoplastic action of DRB18 has not been reported for any hematological malignancies. The wide range of low to high IC50 values indicates the need for testing its efficacy and safety for antineoplastic applications against a wider variety of preclinical cancer models. It is also unclear if DRB18 can modulate the cytotoxic action of other antineoplastic agents. Safety issues on normal tissues and organs of the tumor-bearing host also remain largely unverified. Moreover, DRB18 is water insoluble, which could be another limitation of its use in anticancer strategies.

### BAY-876

A quinoline derivative named BAY-876 (Figure 5C) was initially identified for its highly selective GLUT1 inhibitory potential by (Siebeneicher et al., 2016b), which also exerts its actions in nM ranges but has limited ability to inhibit other GLUT isoforms like GLUT2, GLUT3, and GLUT4. Additional advantages of BAY-876 include its aqueous stability *in vitro* and adequate bioavailability upon administration through the oral route (Siebeneicher et al., 2016b). BAY-876 manifests antineoplastic action mainly *via* its ability to inhibit GLUT1 leading to multiple actions on neoplastic cells, including suppression of proliferation, drug-resistance, EMT, and metabolism (Yang et al., 2021). BAY-876 was reported to manifest increased NADP^+^/NADPH simulating glucose starvation conditions (Chen et al., 2022). However, the antineoplastic activity of BAY-876 needs to be validated on a broader range of neoplastic cells. However, docking studies have been performed on BAY-876 for binding to GLUT1 (Ojelabi, 2017), and its exclusive binding to only GLUT1 limits its therapeutic applications. Moreover, the safety of its *in vivo* applications needs further verification.

### Fasentin

Fasentin N-(4-chloro-3-(trifluoromethyl)phenyl)-3-oxobutanamide (Figure 5D) is a small molecule inhibitor of GLUT that was initially reported as a sensitizer to induction of cell death *via* FAS and TNF (Kraus et al., 2018; Ocaña et al., 2020) later it was reported that Fasentin leads to glucose deprivation by its ability to bind to GLUT1 and GLUT4 as revealed by docking studies (Wood et al., 2008). However, despite the promising GLUT1 inhibitory potential alongside sensitization of cells to death stimuli, not much has been worked out concerning its antineoplastic potential. A recent study reported that fasentin could inhibit the proliferation of endothelial cells and hence may have an implication in antiangiogenic therapeutic strategies. However, its effect on other normal healthy cells must be explored to predict safety issues (Ocaña et al., 2020). Moreover, the effect of fasentin is apparent in about 100 µM range (Ocaña et al., 2020) which is on the higher side than that desired for an effective and safe therapeutic agent.

### STF-31

Another promising GLUT1 inhibitory agent with antineoplastic potential is STF-31 (Figure 5E) (Chan et al., 2011; Adams et al., 2014; Xintaropoulou et al., 2015; Kraus et al., 2018). It was shown initially to manifest renal cell carcinoma (RCC) specific cytotoxic action (Chan et al., 2011). Additionally, STF-31 has been shown to modulate HIF and target NAMPT to manifest its antineoplastic action (Adams et al., 2014; Xintaropoulou et al., 2015). Moreover, Kraus et al. (2018) reported that the anticancer action of STF-31 is apparent only at higher concentrations, and hence there is a need for further optimization and testing on a broad range of neoplastic target cells both *in vitro* and *in vivo* to explore its dual inhibitory action on GLUT1 and NAMPT. However, the utility of the same will depend on the expression levels of GLUT1 and NAMPT, which may vary in a tumor-to-tumor manner. Moreover, it is also reported that if cancer cells express GLUT2, they can overcome the inhibitory action of STF-31 and that it does not harm normal cells (Chan et al., 2011).

### Glutor, a Pan-GLUT inhibitor

Recently a pan-GLUT inhibitor named glutor has been demonstrated to exhibit a potent antineoplastic action in the nanomolar concentration range against cancer cells of diverse origins (Reckzeh and Waldmann, 2020a; Reckzeh and Waldmann, 2020b; Temre et al., 2022). Glutor is (S)-6-Methyl-5-(4-morpholinobenzyl)-4-oxo-2-phenyl-N-(pyridin-3-ylmethyl)-4,5,6,7-tetrahydropyrazolo [1,5-a] pyrazine-6-carboxamide, with the empirical formula C_3_H_32_N_6_O_3_, and molecular weight of 536.62 (Figure 5F) (Reckzeh et al., 2019).

Glutor was identified to inhibit GLUT1, GLUT2 and GLUT3 in HCT116 cells (Reckzeh et al., 2019). The inhibition of the uptake of 2DG was determined to have an IC50 value of 11 nM in most sensitive cell lines, including urinary cancer-derived cell lines, which are most glucose-addicted (Lea et al., 2015). Interestingly glutor was found to impart no cytotoxicity on normal healthy peripheral blood mononuclear cells and Institute for medical research-90 (IMR-90) embryonic lung cells (Reckzeh et al., 2019). Further, the elegant study of Reckzeh et al. (2019) also screened the antineoplastic action of glutor on a battery of about 94 cancer cell lines of diverse origins, and it was observed that the antineoplastic action was exerted with IC50 values of less than 100 nM. Moreover, the inhibitory action of glutor was also evaluated in multicellular spheroids of HCT-116 cells (Reckzeh et al., 2019). Interestingly glutor treatment minimized glucose deprivation in a dose-dependent manner. Hence, glutor displays the potential for effectively inhibiting glucose uptake in solid tumors. However, it was also reported that some cell lines like BxPC-3 exhibit resistance to glutor. The sensitivity or resistance to glutor depends on the cancer cells’ metabolic background (Reckzeh et al., 2019). The glutor resistance cell lines can stitch between glycolytic and OXPHOS phenotypes (Reckzeh et al., 2019). Depending on the metabolic make-up of the target cells and selected dose, glutor could usher a complete glucose deprivation condition, even leading to upregulation of GLUT1 and GLUT3 expression due to hypoglycemia. Moreover, it is also likely that by its ability to cause inhibition of GLUT1, GLUT2, and GLUT3, glutor possesses an extraordinary capacity to overcome the rescue and compensatory mechanisms of highly adaptive neoplastic cells. It is also noteworthy that the study of Reckzeh et al. (2019) also indicated that neoplastic cells’ cytostasis and cytotoxicity could be synergistically upregulated by using glutor along with glutaminase inhibitor CB-839.

Thus, the results obtained based on screening of glutor for its antineoplastic action on 94 cancer cell lines derived from highly malignant cancers revealed the extraordinary ability of glutor to inhibit glucose uptake, glycolytic flux, and cell survival (Pliszka and Szablewski, 2021).

However, despite the evaluation and certification of the antineoplastic action of glutor on cancer cell lines of diverse origins, the same has not been evaluated in any *in vivo* tumor model, which will be necessary to assess the translational value of glutor’s antineoplastic potential for therapeutic applications. Moreover, the issues of bioavailability and ideal administration routes also need to be assessed in an appropriate *in vivo* model.

Glutor treatment of tumor cells inhibited cell survival and increased cell death. It also caused a decrease in glucose uptake associated with altered expression of GLUT1 and GLUT3. HIF-1α, HK-2, LDH-A, and MCT1 also decreased with diminished lactate production and deregulated pH homeostasis. Moreover, the expression of cell survival regulatory molecules p53, Hsp70, IL-2 receptor CD25, and C-myc was modulated upon treatment with glutor. Additionally, glutor treatment triggered mitochondrial membrane depolarization, accompanied by high ROS generation and the modulated ratio of Bcl-2/BAX. Chemosensitivity of tumor cells increased following exposure to glutor and decreased MDR1 expression (Temre et al., 2022). Hence, these observations indicate that glutor has the potential to be used in antineoplastic therapeutic applications.

### Other inhibitors

Other crucial small molecule inhibitors of GLUT include GLUTi1 and GLUTi2 (Kapoor et al., 2016), compound 3 (Siebeneicher et al., 2016a), compound 15b (Liu et al., 2020), PUG1 (Ung et al., 2016), KL-11743 (Olszewski et al., 2022), and NV5440 (S. A. Kang et al., 2019) which belong to diverse families of chemicals and exert limited GLUT isoform inhibitory potential and have high IC50 values, so the possibilities of *in vivo* toxicity remains a concerning issue. Further, the neoplastic cell inhibitory potential in most of these remains limited to only a few targets investigated. Tuccinardi et al. reported the GLUT1 inhibitory potential of oxime-based inhibitors with compatibility to inhibiting the growth of the H1299 lung cancer cell line (Tuccinardi et al., 2013). However, not much has been reported further on the anticancer potential of this category of inhibitors except for their utility in developing novel GLUT inhibitory agents.

### Combinatorial approaches

Studies have indicated the promising potential of a combinatorial approach for GLUT inhibition instead of a single inhibitor approach (Tilekar et al., 2020). Such approaches mainly involve a combination of agents with 1) the ability to directly bind to GLUT isoforms and cause their inhibition; 2) approaches to inhibit expression of GLUT, and 3) use of agents which compete with glucose for binding to GLUTs and hence cause inhibited glucose transport. Further to achieve success in such approaches, GLUT inhibitors with pan-GLUT inhibitory potential can be of great use to usher the inhibition of multiple GLUT isoforms. Moreover, a combination of multiple GLUT inhibitors of natural and synthetic origin can also be considered and has shown hope during *in vitro* experiments on cancer cell lines of various origins ((Kast et al., 2016; Sawayama et al., 2019; Tilekar et al., 2020). However, adding several inhibitors together may cause toxicity and can likely lead to certain harmful effects on normal healthy cells, which also use GLUT isoforms for glucose uptake. Further, most of such combinations still lack the much-required *in vivo* testing for evaluating efficacy and safety parameters. Moreover, WZB117 (Chen et al., 2017), 2DG (Lee et al., 2016), and BAY876 (Mori et al., 2019) have also been sporadically used in combination with chemotherapeutic agents under *in vivo* experimentation. Moreover, several studies have been conducted to evaluate the outcome of combining various GLUT inhibitors for achieving optimum inhibition of glucose uptake (Tilekar et al., 2020). The most promising of such combinations with successful antineoplastic outcomes include combination of GLUT inhibitors like cytochalasin B, WZB117 and its derivatives, BAY-876, silibinin, glutor, STF-31 and phloretin with other conventional anticancer drugs like doxorubicin, curcumin, etoposide, cisplatin, 5-fluorouracil, vincristine, cytarabine, oxaliplatin, paclitaxel and antimycin A (Liu et al., 2012; Tsakalozou et al., 2012; Li et al., 2019; Trendowski, 2015; Trendowski, 2015; Kapoor et al., 2016; Chen et al., 2017; Reckzeh et al., 2019; Zambrano et al., 2019; Barbosa and Martel, 2020; Joly et al., 2020; Tilekar et al., 2020; Wu et al., 2020; Shriwas et al., 2021; Olszewski et al., 2022; Temre et al., 2022; Weng et al., 2022) to name a few such representative anticancer drugs in cancers of diverse origins. Nevertheless, inhibitors of other molecules and/or pathways have also been combined with GLUT inhibitors to achieve fruitful antineoplastic effects (Tsakalozou et al., 2012; Tilekar et al., 2020; Weng et al., 2022). For example, the combination of glutor with glutaminase inhibitor CB-839 resulted in successful antineoplastic manifestations (Reckzeh et al., 2019). Additionally, combination with GLUT inhibitors with radiotherapeutic strategies had also displayed improved antineoplastic potential (Zhao et al., 2016). Moreover, GLUT inhibitors, combined with other antiglycolytic agents, also result in superior inhibition of tumor cell metabolism, compared to such action of these agents when applied alone (Raez et al., 2013; Barbosa and Martel, 2020; Tilekar et al., 2020). These studies are also limited in their long-lasting impact in designing novel therapeutics due to the lack of extensive in vivo-based investigations to assess safety and toxicity issues on normal healthy cells and tissues. However, despite the limitations above, combinatorial approaches hold promise for formidable antineoplastic therapeutic applications to overcome drug- and radio-resistance and synergize with the anticancer action of individual agents. Importantly, a combination of GLUT inhibitors with chemotherapeutic agents will also aid in reducing the cytotoxic dose of chemotherapeutic agents and hence minimize their side effects, which need experimental validation. Moreover, combining GLUT inhibitors with other antineoplastic therapeutic agents to manifest a robust and multifaceted inhibition of tumor metabolism could also be a promising antineoplastic strategy. However, such possible combinations are yet to be fully explored. The combinatorial approach of multiagent strategies must be carefully explored to optimize anticancer action while minimizing the side effect. These promising results are also being explored for translational therapeutic potential in clinical trials.

## Additional approaches

### GLUT antibodies

For a long, attempts have been made to make the best utilization of anti-GLUT antibodies to block glucose transport by GLUT. Anti-GLUT1 antibodies have been demonstrated to inhibit glucose uptake in resealed erythrocyte membrane ghosts (Afzal et al., 2004). Nevertheless, a study using Cal27 cells showed that anti-GLUT1 antibodies could inhibit proliferation, induce apoptosis, and chemosensitization for cisplatin (Wang et al., 2013). A study by Rastogi et al. (2007) reported that anti-GLUT1 antibodies could arrest cell growth accompanied by induction of apoptosis in cell lines derived from breast and non-small cell lung cancer (NSCLC). Additionally, anti-GLUT1 antibodies also chemosensitized MCF7 cells to cisplatin, paclitaxel, and gefitinib (Rastogi et al., 2007). Further, anti-GLUT1 antibodies have also been explored for the targeted delivery of chemotherapeutic agents (Barbosa and Martel, 2020). However, a literature search reveals that the spectrum of cancers against which anti GLUT1 antibodies have been tested is limited to realizing their therapeutic potential.

### miRNAs in anti-GLUT strategies

There have been attempts to use the potential of miRNAs to target various aspects of tumor metabolism, including GLUTs (Chen et al., 2012; Pedroza-Torres et al., 2019; Shiah et al., 2021). miRNAs like miR-133 and miR-195–5P have modulated gene transcription of various GLUT isoforms (Macheda et al., 2005; Krüger et al., 2008). In this quest, miRNA to GLUT1, GLUT3, and GLUT4 has shown promising antineoplastic potential in prevalent cancers like lung, prostate, breast, colorectal carcinoma, bladder cancer, and pancreatic adenocarcinoma (Lu et al., 2010; Chen et al., 2012; Fei et al., 2012; Chen et al., 2015; Zhao et al., 2017; Pedroza-Torres et al., 2019; Azizi et al., 2021; Shiah et al., 2021). Moreover, a direct inverse correlation has been reported between miRNA-144 and GLUT1 in breast and non-small cell lung cancer (NSCLC) (Azizi et al., 2021). Similarly, the role of miR-22, miR1291, and miR-195–5P have been associated with GLUT1, and GLUT3 in breast, RCC, and T24 cells, respectively (Fei et al., 2012; Yamasaki et al., 2013; Chen et al., 2015; Alamoudi et al., 2018). Given such intimacy between various miRNAs and GLUT isoforms, there is a strong logic to utilize them in therapeutic applications. Similarly, a study on the role of MiR-218 and MiR340 in the expression of GLUT1 is demonstrated in oral squamous cell carcinoma (OSCC) (Xu et al., 2018; Wang et al., 2020; Shiah et al., 2021). Combinatorial use of miRNAs with other conventional GLUT1 inhibitors is also needed to be explored to circumvent GLUT at both gene expression and protein function levels.

### Short hairpin RNA

Short hairpin RNA (shRNA) to GLUT1 has shown a promising inhibitory effect on M.D. Anderson - Metastatic Breast 231 (MDA-MB-231) and *Homo sapiens*-578 tumor cells (HS578T) triple-negative breast cancer (TNBC) cell lines (Oh et al., 2017; Shriwas et al., 2018; Wu et al., 2020). Similarly, the use of shRNA to GLUT1 was observed to show promising outcomes in colon cancer cells (Bai et al., 2019). Wang et al. (2020) demonstrated that shRNA inhibits the survival of laryngeal carcinoma HEP2 cells *via* beclin-1-associated autophagy. shRNA to GLUT1 is also reported to effectively block glucose uptake in various cancer cell lines (Pliszka and Szablewski, 2021). Thus, the shRNA approach is also promising for effective combat against GLUT expression and function in neoplastic cells by silencing gene expression of various isoforms of glucose transporters.

### Antisense cDNA

Approaches to transfecting neoplastic cells with GLUT1 antisense cDNA have been demonstrated to usher in a decline in the gene expression of GLUT1 and an increase in radiosensitivity of neoplastic cells (Chan et al., 1999; Ito et al., 2000; Yan et al., 2013; Pliszka and Szablewski, 2021). The common cancers used to test this approach include leukemia, gastric, rhabdosarcoma, breast, and glioblastoma (Ito et al., 2002; Chan et al., 2004; Yang et al., 2019; Pliszka and Szablewski, 2021). However, not much breakthrough is yet reported using this approach, and its clinical applications need to be evaluated. The approaches mentioned above of ‘nipping in the bud’ of GLUT expression level have still not come into vogue for preclinical and clinical trials as more basic data needs to be collected before embarking further. However, the most popular and explored approaches are still to identify the best pan-GLUT inhibitor, as other approaches to gene intervention can have potentially harmful outcomes. In this quest, a newly recognized pan-GLUT inhibitor named glutor is emerging with the hope of effective GLUT1 and GLUT3 inhibition.

## Epilogue

**FIGURE 6:**
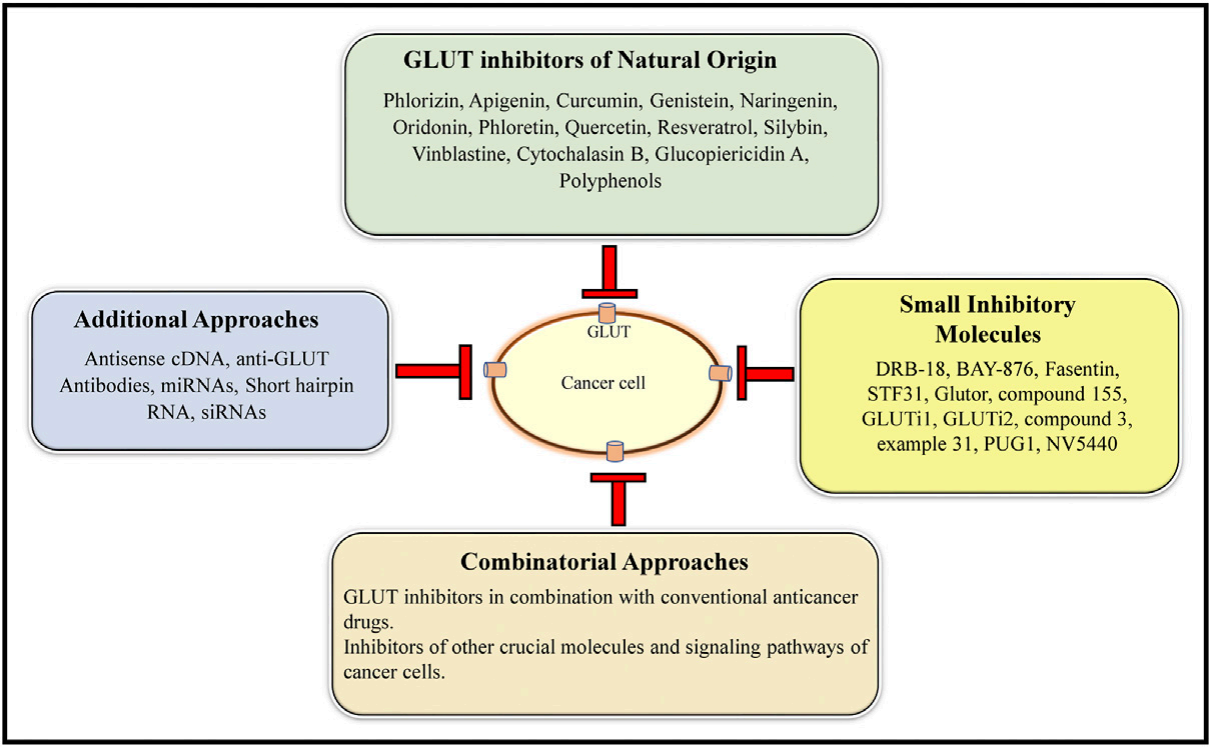
A collation of the current GLUT inhibitory approaches. The main GLUT inhibitors belong to both natural and small inhibitory molecule categories. In addition, antisense cDNA, anti-GLUT antibodies, miRNAs, shRNA, and siRNAs constitute additional GLUT inhibitory approaches. GLUT inhibition can be further potentiated by combining the GLUT inhibitors of natural origin and small inhibitory molecule categories.

The review above shows that inhibition of GLUT can be a promising antineoplastic therapeutic approach, which needs to be vigorously approached at preclinical and clinical levels. Figure 6 shows a summary of the various GLUT inhibition strategies holding a strong supporting experimental evidence. Since many of these strategies have been explored only on cell lines, their further validation in appropriate *in vivo* animal models is essential before exploring the clinical potential. As stated above, selecting those GLUT inhibitors with a pan-GLUT inhibitory potential at low concentrations will be necessary to avoid toxicity on normal healthy cells and organs. Assessing safety on crucial physiological parameters, including blood profile, renal and hepatic function, will also be essential. The half-life of the GLUT inhibitors and their renal clearance needs to be determined for dose optimization. As GLUT plays a critical role in neurological functions, the ability of GLUT inhibitors to cross the blood-brain barrier must be critically evaluated.

It will be essential to consider the safety of the combinatorial approach of GLUT inhibitors. As a massive fraction of the human population is coming under various metabolic disorders, including diabetes, GLUT inhibitors’ impact must be carefully evaluated in such patients. However, overcoming these lacunas and unaddressed issues indicates a bright future for using GLUT inhibition approaches in anticancer regimens.

Based on the criteria for selection of GLUT inhibitors, it emerges that glutor is one of the best to choose for further investigations owing to the following points in its favor:

1) It is worked out to be a Pan-GLUT inhibitor and hence is supposed to exert a more potent inhibitory action on glucose uptake
2) It has been screened against 94 cancer cell lines and is found to be effective in more than half of cell lines for its cytostatic action, and no other small molecule inhibitor has been tested on such a broad range of target cancer cells of diverse etiology.
3) Its antineoplastic action is exerted in the nanomolar concentration range and has been shown to have the least toxic effect on normal cells.
4) Its easy accessibility at the commercial level for further investigation.

Therefore, based on our evaluation glutor has an edge over other pan-GLUT inhibitors for further investigations at preclinical and clinical level.

## References

[B1] Actis Dato V. Sánchez M. C. Chiabrando G. A. (2021). LRP1 mediates the IGF-1-induced GLUT1 expression on the cell surface and glucose uptake in Müller glial cells. Sci. Rep. 11, 4742. 10.1038/s41598-021-84090-3 33637845 PMC7910306

[B2] Adams D. J. Ito D. Rees M. G. Seashore-Ludlow B. Puyang X. Ramos A. H. (2014). NAMPT is the cellular target of STF-31-like small-molecule probes. ACS Chem. Biol. 9, 2247–2254. 10.1021/cb500347p 25058389 PMC4201331

[B3] Adekola K. Rosen S. T. Shanmugam M. (2012). Glucose transporters in cancer metabolism. Curr. Opin. Oncol. 24, 650–654. 10.1097/CCO.0b013e328356da72 22913968 PMC6392426

[B4] Afzal I. Browning J. A. Drew C. Ellory J. C. Naftalin R. J. Wilkins R. J. (2004). Effects of anti-GLUT antibodies on glucose transport into human erythrocyte ghosts. Bioelectrochemistry 62, 195–198. 10.1016/j.bioelechem.2003.07.007 15039027

[B5] Alamoudi A. A. Alnoury A. Gad H. (2018). miRNA in tumour metabolism and why could it be the preferred pathway for energy reprograming. Brief. Funct. Genomics 17, 157–169. 10.1093/bfgp/elx023 29028873

[B6] Almahmoud S. Wang X. Vennerstrom J. L. Zhong H. A. (2019). Conformational studies of glucose transporter 1 (GLUT1) as an anticancer drug target. Molecules 24, 2159. 10.3390/molecules24112159 PMC6600248 31181707

[B7] Alvarez J. Lee D. C. Baldwin S. A. Chapman D. (1987). Fourier transform infrared spectroscopic study of the structure and conformational changes of the human erythrocyte glucose transporter. J. Biol. Chem. 262, 3502–3509. 10.1016/S0021-9258(18)61379-1 3818652

[B8] Alzaid F. Cheung H.-M. Preedy V. R. Sharp P. A. (2013). Regulation of glucose transporter expression in human intestinal caco-2 cells following exposure to an anthocyanin-rich berry extract. PLoS ONE 8, e78932. 10.1371/journal.pone.0078932 24236070 PMC3827299

[B9] Ancey P.-B. Contat C. Meylan E. (2018). Glucose transporters in cancer – from tumor cells to the tumor microenvironment. FEBS J. 285, 2926–2943. 10.1111/febs.14577 29893496

[B10] Andrianifahanana M. Hernandez D. M. Yin X. Kang J.-H. Jung M.-Y. Wang Y. (2016). Profibrotic up-regulation of glucose transporter 1 by TGF-β involves activation of MEK and mammalian target of rapamycin complex 2 pathways. FASEB J. 30, 3733–3744. 10.1096/fj.201600428R 27480571 PMC5067255

[B11] Azizi M. I. H. N. Othman I. Naidu R. (2021). The role of MicroRNAs in lung cancer metabolism. Cancers 13, 1716. 10.3390/cancers13071716 33916349 PMC8038585

[B12] Bai J. Xu J. Zhao J. Zhang R. (2019). Downregulation of lncRNA AWPPH inhibits colon cancer cell proliferation by downregulating GLUT-1. Oncol. Lett. 18, 2007–2012. 10.3892/ol.2019.10515 31423271 PMC6614671

[B13] Bakht M. K. Lovnicki J. M. Tubman J. Stringer K. F. Chiaramonte J. Reynolds M. R. (2020). Differential expression of glucose transporters and hexokinases in prostate cancer with a neuroendocrine gene signature: a mechanistic perspective for ^18^F-FDG imaging of PSMA-suppressed tumors. J. Nucl. Med. 61, 904–910. 10.2967/jnumed.119.231068 31806771 PMC7262227

[B14] Barbosa A. M. Martel F. (2020). Targeting glucose transporters for breast cancer therapy: the effect of natural and synthetic compounds. Cancers 12, 154. 10.3390/cancers12010154 PMC7016663 31936350

[B15] Barron C. C. Bilan P. J. Tsakiridis T. Tsiani E. (2016). Facilitative glucose transporters: Implications for cancer detection, prognosis and treatment. Metabolism. 65, 124–139. 10.1016/j.metabol.2015.10.007 26773935

[B16] Barron C. Tsiani E. Tsakiridis T. (2012). Expression of the glucose transporters GLUT1, GLUT3, GLUT4 and GLUT12 in human cancer cells. BMC Proc. 6, P4. 10.1186/1753-6561-6-S3-P4

[B17] Boado R. J. Black K. L. Pardridge W. M. (1994). Gene expression of GLUT3 and GLUT1 glucose transporters in human brain tumors. Brain Res. Mol. Brain Res. 27, 51–57. 10.1016/0169-328x(94)90183-x 7877454

[B18] Brosius F. C. Briggs J. P. Marcus R. G. Barac-Nieto M. Charron M. J. (1992). Insulin-responsive glucose transporter expression in renal microvessels and glomeruli. Kidney Int. 42, 1086–1092. 10.1038/ki.1992.391 1453596

[B19] Brunetto E. L. Teixeira S. da S. Giannocco G. Machado U. F. Nunes M. T. (2012). T3 rapidly increases SLC2A4 gene expression and GLUT4 trafficking to the plasma membrane in skeletal muscle of rat and improves glucose homeostasis. Thyroid 22, 70–79. 10.1089/thy.2010.0409 22136156

[B20] Burant C. F. Bell G. I. (1992). Mammalian facilitative glucose transporters: evidence for similar substrate recognition sites in functionally monomeric proteins. Biochemistry 31, 10414–10420. 10.1021/bi00157a032 1420159

[B21] Calvo M. B. Figueroa A. Pulido E. G. Campelo R. G. Aparicio L. A. (2010). Potential role of sugar transporters in cancer and their relationship with anticancer therapy. Int. J. Endocrinol. 2010, e205357. 10.1155/2010/205357 PMC2913528 20706540

[B22] Carvalho K. C. Cunha I. W. Rocha R. M. Ayala F. R. Cajaíba M. M. Begnami M. D. (2011). GLUT1 expression in malignant tumors and its use as an immunodiagnostic marker. Clinics 66, 965–972. 10.1590/S1807-59322011000600008 21808860 PMC3129958

[B23] Cassim S. Vučetić M. Ždralević M. Pouyssegur J. (2020). Warburg and beyond: The power of mitochondrial metabolism to collaborate or replace fermentative glycolysis in cancer. Cancers 12, 1119. 10.3390/cancers12051119 PMC7281550 32365833

[B24] Chadt A. Al-Hasani H. (2020). Glucose transporters in adipose tissue, liver, and skeletal muscle in metabolic health and disease. Pflugers Arch. 472, 1273–1298. 10.1007/s00424-020-02417-x 32591906 PMC7462924

[B25] Chan D. A. Sutphin P. D. Nguyen P. Turcotte S. Lai E. W. Banh A. (2011). Targeting GLUT1 and the Warburg effect in renal cell carcinoma by chemical synthetic lethality. Sci. Transl. Med. 3, 94ra70. 10.1126/scitranslmed.3002394 PMC3683134 21813754

[B26] Chan J. Y. Kong S. K. Choy Y. M. Lee C. Y. Fung K. P. (1999). Inhibition of glucose transporter gene expression by antisense nucleic acids in HL-60 leukemia cells. Life Sci. 65, 63–70. 10.1016/s0024-3205(99)00219-2 10403494

[B27] Chan K. K. Chan J. Y. W. Chung K. K. W. Fung K.-P. (2004). Inhibition of cell proliferation in human breast tumor cells by antisense oligonucleotides against facilitative glucose transporter 5. J. Cell. Biochem. 93, 1134–1142. 10.1002/jcb.20270 15449313

[B28] Chen B. Li H. Zeng X. Yang P. Liu X. Zhao X. (2012). Roles of microRNA on cancer cell metabolism. J. Transl. Med. 10, 228. 10.1186/1479-5876-10-228 23164426 PMC3563491

[B29] Chen B. Tang H. Liu X. Liu P. Yang L. Xie X. (2015). miR-22 as a prognostic factor targets glucose transporter protein type 1 in breast cancer. Cancer Lett. 356, 410–417. 10.1016/j.canlet.2014.09.028 25304371

[B30] Chen H. Zhang H. Cao L. Cui J. Ma X. Zhao C. (2022). Glucose limitation sensitizes cancer cells to selenite-induced cytotoxicity via SLC7A11-mediated redox collapse. Cancers 14, 345. 10.3390/cancers14020345 35053507 PMC8773648

[B31] Chen Q. Meng Y.-Q. Xu X.-F. Gu J. (2017). Blockade of GLUT1 by WZB117 resensitizes breast cancer cells to adriamycin. Anticancer. Drugs 28, 880–887. 10.1097/CAD.0000000000000529 28609310

[B32] Custódio T. F. Paulsen P. A. Frain K. M. Pedersen B. P. (2021). Structural comparison of GLUT1 to GLUT3 reveal transport regulation mechanism in sugar porter family. Life Sci. Alliance 4, e202000858. 10.26508/lsa.202000858 33536238 PMC7898563

[B33] de Blas E. Estañ M. C. del Carmen Gómez de Frutos M. Ramos J. del Carmen Boyano-Adánez M. Aller P. (2016). Selected polyphenols potentiate the apoptotic efficacy of glycolytic inhibitors in human acute myeloid leukemia cell lines. Regulation by protein kinase activities. Cancer Cell Int. 16, 70. 10.1186/s12935-016-0345-y 27610044 PMC5015235

[B34] De Los Santos F. G. (2013). Regulation of glucose transporter 1 (Slc2a1) in the pituitary gonadotrope of mice after puberty. J. Steroids Horm. Sci. 05. 10.4172/2157-7536.1000138

[B35] Deng D. Sun P. Yan C. Ke M. Jiang X. Xiong L. (2015). Molecular basis of ligand recognition and transport by glucose transporters. Nature 526, 391–396. 10.1038/nature14655 26176916

[B36] Deng D. Xu C. Sun P. Wu J. Yan C. Hu M. (2014). Crystal structure of the human glucose transporter GLUT1. Nature 510, 121–125. 10.1038/nature13306 24847886

[B37] Deng D. Yan N. (2016). GLUT, SGLT, and SWEET: Structural and mechanistic investigations of the glucose transporters. Protein Sci. 25, 546–558. 10.1002/pro.2858 26650681 PMC4815417

[B38] Devés R. Krupka R. M. (1978). Cytochalasin B and the kinetics of inhibition of biological transport: a case of asymmetric binding to the glucose carrier. Biochim. Biophys. Acta 510, 339–348. 10.1016/0005-2736(78)90034-2 667049

[B39] Diaz-Ruiz R. Rigoulet M. Devin A. (2011). The Warburg and Crabtree effects: On the origin of cancer cell energy metabolism and of yeast glucose repression. Biochim. Biophys. Acta 1807, 568–576. 10.1016/j.bbabio.2010.08.010 20804724

[B40] Ding Y. Tian Y. Guo M. Liu J. Heng D. Zhu B. (2016). Regulation of glucose transport by thyroid hormone in rat ovary. Cell Tissue Res. 366, 455–466. 10.1007/s00441-016-2453-3 27411690

[B41] Drew D. North R. A. Nagarathinam K. Tanabe M. (2021). Structures and general transport mechanisms by the major facilitator superfamily (MFS). Chem. Rev. 121, 5289–5335. 10.1021/acs.chemrev.0c00983 33886296 PMC8154325

[B42] Echeverría C. Nualart F. Ferrada L. Smith G. J. Godoy A. S. (2021). Hexose transporters in cancer: From multifunctionality to diagnosis and therapy. Trends Endocrinol. Metab. 32, 198–211. 10.1016/j.tem.2020.12.006 33518451

[B43] Farrell T. L. Ellam S. L. Forrelli T. Williamson G. (2013). Attenuation of glucose transport across caco-2 cell monolayers by a polyphenol-rich herbal extract: interactions with SGLT1 and GLUT2 transporters. Biofactors 39, 448–456. 10.1002/biof.1090 23361943

[B44] Fei X. Qi M. Wu B. Song Y. Wang Y. Li T. (2012). MicroRNA-195-5p suppresses glucose uptake and proliferation of human bladder cancer T24 cells by regulating GLUT3 expression. FEBS Lett. 586, 392–397. 10.1016/j.febslet.2012.01.006 22265971

[B45] Freemerman A. J. Johnson A. R. Sacks G. N. Milner J. J. Kirk E. L. Troester M. A. (2014). Metabolic reprogramming of macrophages: glucose transporter 1 (GLUT1)-mediated glucose metabolism drives a proinflammatory phenotype. J. Biol. Chem. 289, 7884–7896. 10.1074/jbc.M113.522037 24492615 PMC3953299

[B46] Frolova A. Flessner L. Chi M. Kim S. T. Foyouzi-Yousefi N. Moley K. H. (2009). Facilitative glucose transporter type 1 is differentially regulated by progesterone and estrogen in murine and human endometrial stromal cells. Endocrinology 150, 1512–1520. 10.1210/en.2008-1081 18948400 PMC2654750

[B47] Frolova A. I. Moley K. H. (2011). Quantitative analysis of glucose transporter mRNAs in endometrial stromal cells reveals critical role of GLUT1 in uterine receptivity. Endocrinology 152, 2123–2128. 10.1210/en.2010-1266 21343253 PMC3075937

[B48] Gaber G. El Achy S. Khedr G. A. Parimi V. Helenowksi I. Donnelly E. D. (2021). Impact of p53, HIF1a, ki-67, CA-9, and GLUT1 expression on treatment outcomes in locally advanced cervical cancer patients treated with definitive chemoradiation therapy. Am. J. Clin. Oncol. 44, 58–67. 10.1097/COC.0000000000000781 33284239

[B49] Galochkina T. Ng Fuk Chong M. Challali L. Abbar S. Etchebest C. (2019). New insights into GluT1 mechanics during glucose transfer. Sci. Rep. 9, 998. 10.1038/s41598-018-37367-z 30700737 PMC6353926

[B51] George Thompson A. M. Iancu C. V. Nguyen T. T. H. Kim D. Choe J. (2015). Inhibition of human GLUT1 and GLUT5 by plant carbohydrate products; insights into transport specificity. Sci. Rep. 5, 12804. 10.1038/srep12804 26306809 PMC4549712

[B52] Ghanavat M. Shahrouzian M. Deris Zayeri Z. Banihashemi S. Kazemi S. M. Saki N. (2021). Digging deeper through glucose metabolism and its regulators in cancer and metastasis. Life Sci. 264, 118603. 10.1016/j.lfs.2020.118603 33091446

[B53] Gillies R. J. Gatenby R. A. (2007). Adaptive landscapes and emergent phenotypes: why do cancers have high glycolysis? J. Bioenerg. Biomembr. 39, 251–257. 10.1007/s10863-007-9085-y 17624581

[B54] Godoy A. Ulloa V. Rodríguez F. Reinicke K. Yañez A. J. García M. (2006). Differential subcellular distribution of glucose transporters GLUT1–6 and GLUT9 in human cancer: Ultrastructural localization of GLUT1 and GLUT5 in breast tumor tissues. J. Cell. Physiol. 207, 614–627. 10.1002/jcp.20606 16523487

[B55] Gonzalez-Menendez P. Hevia D. Rodriguez-Garcia A. Mayo J. C. Sainz R. M. (2014). Regulation of GLUT transporters by flavonoids in androgen-sensitive and -insensitive prostate cancer cells. Endocrinology 155, 3238–3250. 10.1210/en.2014-1260 24932809

[B56] Granchi C. Fortunato S. Minutolo F. (2016). Anticancer agents interacting with membrane glucose transporters. MedChemComm 7, 1716–1729. 10.1039/C6MD00287K 28042452 PMC5198910

[B57] Gunnink L. K. Alabi O. D. Kuiper B. D. Gunnink S. M. Schuiteman S. J. Strohbehn L. E. (2016). Curcumin directly inhibits the transport activity of GLUT1. Biochimie 125, 179–185. 10.1016/j.biochi.2016.03.014 27039889 PMC5006061

[B58] Gwak H. Haegeman G. Tsang B. K. Song Y. S. (2015). Cancer-specific interruption of glucose metabolism by resveratrol is mediated through inhibition of Akt/GLUT1 axis in ovarian cancer cells. Mol. Carcinog. 54, 1529–1540. 10.1002/mc.22227 25307508

[B59] Hall C. Wu M. Crane F. L. Takahashi H. Tamura S. Folkers K. (1966). Piericidin A: a new inhibitor of mitochondrial electron transport. Biochem. Biophys. Res. Commun. 25, 373–377. 10.1016/0006-291x(66)90214-2 4290528

[B60] Hamilton K. E. Rekman J. F. Gunnink L. K. Busscher B. M. Scott J. L. Tidball A. M. (2018). Quercetin inhibits glucose transport by binding to an exofacial site on GLUT1. Biochimie 151, 107–114. 10.1016/j.biochi.2018.05.012 29857184 PMC6035882

[B61] Hanahan D. (2022). Hallmarks of cancer: New dimensions. Cancer Discov. 12, 31–46. 10.1158/2159-8290.CD-21-1059 35022204

[B62] Hanahan D. Weinberg R. A. (2011). Hallmarks of cancer: the next generation. Cell 144, 646–674. 10.1016/j.cell.2011.02.013 21376230

[B63] Harris V. M. Bendre S. V. Santos F. G. D. L. Fite A. El-Dandachli A. E.-Y. Kurenbekova L. (2012). GnRH increases glucose transporter-1 expression and stimulates glucose uptake in the gonadotroph. J. Endocrinol. 212, 139–147. 10.1530/JOE-11-0359 22107955

[B64] Hayashi M. Sakata M. Takeda T. Yamamoto T. Okamoto Y. Sawada K. (2004). Induction of glucose transporter 1 expression through hypoxia-inducible factor 1alpha under hypoxic conditions in trophoblast-derived cells. J. Endocrinol. 183, 145–154. 10.1677/joe.1.05599 15525582

[B65] Heinze M. Monden I. Keller K. (2004). Cysteine-scanning mutagenesis of transmembrane segment 1 of glucose transporter GLUT1: Extracellular accessibility of helix positions. Biochemistry 43, 931–936. 10.1021/bi030175w 14744136

[B66] Heydarzadeh S. Moshtaghie A. A. Daneshpour M. Hedayati M. (2022), Molecular mechanisms of glucose uptake regulation in thyroid cancer. In (Ed.), Hypothyroidism - new aspects of an old disease [working title]. London: IntechOpen. 10.5772/intechopen.101937

[B67] Holman G. D. (2020). Structure, function and regulation of mammalian glucose transporters of the SLC2 family. Pflugers Arch. 472, 1155–1175. 10.1007/s00424-020-02411-3 32591905 PMC7462842

[B68] Hoxhaj G. Manning B. D. (2020). The PI3K-AKT network at the interface of oncogenic signalling and cancer metabolism. Nat. Rev. Cancer 20, 74–88. 10.1038/s41568-019-0216-7 31686003 PMC7314312

[B69] Hresko R. C. Kraft T. E. Quigley A. Carpenter E. P. Hruz P. W. (2016). Mammalian glucose transporter activity is dependent upon anionic and conical phospholipids. J. Biol. Chem. 291, 17271–17282. 10.1074/jbc.M116.730168 27302065 PMC5016126

[B70] Hu Y. Yang Z. Bao D. Ni J.-S. Lou J. (2019). miR-455-5p suppresses hepatocellular carcinoma cell growth and invasion via IGF-1R/AKT/GLUT1 pathway by targeting IGF-1R. Pathol. Res. Pract. 215, 152674. 10.1016/j.prp.2019.152674 31732382

[B71] Imoto M. (2019). Chemistry and biology for the small molecules targeting characteristics of cancer cells. Biosci. Biotechnol. Biochem. 83, 10–19. 10.1080/09168451.2018.1518704 30247093

[B72] Ito S. Fukusato T. Nemoto T. Sekihara H. Seyama Y. Kubota S. (2002). Coexpression of glucose transporter 1 and matrix metalloproteinase-2 in human cancers. J. Natl. Cancer Inst. 94, 1080–1091. 10.1093/jnci/94.14.1080 12122099

[B73] Ito S. Nemoto T. Satoh S. Sekihara H. Seyama Y. Kubota S. (2000). Human rhabdomyosarcoma cells retain insulin-regulated glucose transport activity through glucose transporter 1. Arch. Biochem. Biophys. 373, 72–82. 10.1006/abbi.1999.1535 10620325

[B74] Ji J. Yang X. Flavel M. Shields Z. P.-I. Kitchen B. (2019). Antioxidant and anti-diabetic functions of a polyphenol-rich sugarcane extract. J. Am. Coll. Nutr. 38, 670–680. 10.1080/07315724.2019.1587323 31008696

[B75] Johnston K. Sharp P. Clifford M. Morgan L. (2005). Dietary polyphenols decrease glucose uptake by human intestinal Caco-2 cells. FEBS Lett. 579, 1653–1657. 10.1016/j.febslet.2004.12.099 15757656

[B76] Joly J. H. Delfarah A. Phung P. S. Parrish S. Graham N. A. (2020). A synthetic lethal drug combination mimics glucose deprivation–induced cancer cell death in the presence of glucose. J. Biol. Chem. 295, 1350–1365. 10.1074/jbc.RA119.011471 31914417 PMC6996897

[B77] Joost H.-G. Bell G. I. Best J. D. Birnbaum M. J. Charron M. J. Chen Y. T. (2002). Nomenclature of the GLUT/SLC2A family of sugar/polyol transport facilitators. Am. J. Physiol. Endocrinol. Metab. 282, E974–E976. 10.1152/ajpendo.00407.2001 11882521

[B78] Joost H.-G. Thorens B. (2001). The extended GLUT-family of sugar/polyol transport facilitators: nomenclature, sequence characteristics, and potential function of its novel members (review). Mol. Membr. Biol. 18, 247–256. 10.1080/09687680110090456 11780753

[B79] Jung C. Y. Rampal A. L. (1977). Cytochalasin B binding sites and glucose transport carrier in human erythrocyte ghosts. J. Biol. Chem. 252, 5456–5463. 10.1016/s0021-9258(19)63372-7 885863

[B80] Jung C. Y. (1996). The facilitative glucose transporter and insulin action. Exp. Mol. Med. 28, 153–160. 10.1038/emm.1996.24

[B81] Jung K.-H. Lee J. H. Thien Quach C. H. Paik J.-Y. Oh H. Park J. W. (2013). Resveratrol suppresses cancer cell glucose uptake by targeting reactive oxygen species-mediated hypoxia-inducible factor-1α activation. J. Nucl. Med. 54, 2161–2167. 10.2967/jnumed.112.115436 24221993

[B82] Kang G. G. Francis N. Hill R. Waters D. Blanchard C. Santhakumar A. B. (2019). Dietary polyphenols and gene expression in molecular pathways associated with type 2 diabetes mellitus: A review. Int. J. Mol. Sci. 21, 140. 10.3390/ijms21010140 PMC6981492 31878222

[B83] Kang S. A. O’Neill D. J. Machl A. W. Lumpkin C. J. Galda S. N. Sengupta S. (2019). Discovery of small-molecule selective mTORC1 inhibitors via direct inhibition of glucose transporters. Cell Chem. Biol. 26, 1203–1213. 10.1016/j.chembiol.2019.05.009 31231029

[B84] Kapoor K. Finer-Moore J. S. Pedersen B. P. Caboni L. Waight A. Hillig R. C. (2016). Mechanism of inhibition of human glucose transporter GLUT1 is conserved between cytochalasin B and phenylalanine amides. Proc. Natl. Acad. Sci. U. S. A. 113, 4711–4716. 10.1073/pnas.1603735113 27078104 PMC4855560

[B85] Kast R. E. Ramiro S. Lladó S. Toro S. Coveñas R. Muñoz M. (2016). Antitumor action of temozolomide, ritonavir and aprepitant against human glioma cells. J. Neurooncol. 126, 425–431. 10.1007/s11060-015-1996-6 26603162

[B86] Keating E. Martel F. (2018). Antimetabolic effects of polyphenols in breast cancer cells: Focus on glucose uptake and metabolism. Front. Nutr. 5, 25. 10.3389/fnut.2018.00025 29713632 PMC5911477

[B87] Kido T. Murata H. Nishigaki A. Tsubokura H. Komiya S. Kida N. (2020). Glucose transporter 1 is important for the glycolytic metabolism of human endometrial stromal cells in hypoxic environment. Heliyon 6, e03985. 10.1016/j.heliyon.2020.e03985 32548315 PMC7286975

[B88] Kierans S. J. Taylor C. T. (2021). Regulation of glycolysis by the hypoxia-inducible factor (HIF): implications for cellular physiology. J. Physiol. 599, 23–37. 10.1113/JP280572 33006160

[B89] Kilgour E. Baldwin S. A. Flint D. J. (1995). Divergent regulation of rat adipocyte GLUT1 and GLUT4 glucose transporters by GH. J. Endocrinol. 145, 27–33. 10.1677/joe.0.1450027 7798027

[B90] Kim S.-H. Park M.-J. (2017). Effects of growth hormone on glucose metabolism and insulin resistance in human. Ann. Pediatr. Endocrinol. Metab. 22, 145–152. 10.6065/apem.2017.22.3.145 29025199 PMC5642081

[B91] Kitagawa M. Ikeda S. Tashiro E. Soga T. Imoto M. (2010). Metabolomic identification of the target of the filopodia protrusion inhibitor glucopiericidin A. Chem. Biol. 17, 989–998. 10.1016/j.chembiol.2010.06.017 20851348

[B92] Klip A. Pâquet M. R. (1990). Glucose transport and glucose transporters in muscle and their metabolic regulation. Diabetes Care 13, 228–243. 10.2337/diacare.13.3.228 2407478

[B93] Kong X.-Z. Hu S.-S. Sun Z. Zuo L.-H. Kang J. Zhu Z.-F. (2016). Regulation of aerobic glycolysis by long non-coding RNAs in cancer. Biochem. Biophys. Res. Commun. 479, 28–32. 10.1016/j.bbrc.2016.09.007 27596968

[B94] Kraus D. Reckenbeil J. Veit N. Kuerpig S. Meisenheimer M. Beier I. (2018). Targeting glucose transport and the NAD pathway in tumor cells with STF-31: a re-evaluation. Cell. Oncol. 41, 485–494. 10.1007/s13402-018-0385-5 PMC12995222 29949049

[B95] Krüger M. Moser M. Ussar S. Thievessen I. Luber C. A. Forner F. (2008). SILAC mouse for quantitative proteomics uncovers kindlin-3 as an essential factor for red blood cell function. Cell 134, 353–364. 10.1016/j.cell.2008.05.033 18662549

[B96] Krzeslak A. Wojcik-Krowiranda K. Forma E. Jozwiak P. Romanowicz H. Bienkiewicz A. (2012). Expression of GLUT1 and GLUT3 glucose transporters in endometrial and breast cancers. Pathol. Oncol. Res. 18, 721–728. 10.1007/s12253-012-9500-5 22270867 PMC3342495

[B97] Kwon O. Levine M. (2007). Inhibition of the intestinal glucose transporter. GLUT2 by flavonoids 21, 366–377. 10.1096/fj.06-6620com 17172639

[B98] Lea M. A. Altayyar M. desBordes C. (2015). Inhibition of growth of bladder cancer cells by 3-(3-Pyridinyl)-1-(4-pyridinyl)-2-propen-1-one in combination with other compounds affecting glucose metabolism. Anticancer Res. 35, 5889–5899. 26504012

[B99] Lee E. E. Ma J. Sacharidou A. Mi W. Salato V. K. Nguyen N. (2015). A Protein Kinase C phosphorylation motif in GLUT1 affects glucose transport and is mutated in GLUT1 deficiency syndrome. Mol. Cell 58, 845–853. 10.1016/j.molcel.2015.04.015 25982116 PMC4458224

[B100] Lee S.-H. Golinska M. Griffiths J. R. (2021). HIF-1-Independent mechanisms regulating metabolic adaptation in hypoxic cancer cells. Cells 10, 2371. 10.3390/cells10092371 34572020 PMC8472468

[B101] Lee S. J. Park B.-N. Roh J. H. An Y.-S. Hur H. Yoon J.-K. (2016). Enhancing the therapeutic efficacy of 2-deoxyglucose in breast cancer cells using cell-cycle synchronization. Anticancer Res. 36, 5975–5980. 10.21873/anticanres.11185 27793923

[B102] LeFevre P. G. (1948). Evidence of active transfer of certain non-electrolytes across the human red cell membrane. J. Gen. Physiol. 31, 505–527. 10.1085/jgp.31.6.505 18870870 PMC2147122

[B103] Li N. Tan W. Li J. Li P. Lee S. Wang Y. (2011). Glucose metabolism in breast cancer and its implication in cancer therapy. Int. J. Clin. Med. 2, 110–128. 10.4236/ijcm.2011.22022

[B104] Li Y. Zhao L. Li X.-F. (2021). Hypoxia and the tumor microenvironment. Technol. Cancer Res. Treat. 20, 15330338211036304. 10.1177/15330338211036304 34350796 PMC8358492

[B246] Li Y.-L. Weng H.-C. Hsu J.-L. Lin S.-W. Guh J.-H. Hsu L.-C. (2019). The combination of MK-2206 and WZB117 exerts a synergistic cytotoxic effect against breast cancer cells. Front. Pharmacol. 10, 1311. 10.3389/fphar.2019.01311 31780937 PMC6856645

[B105] Lin S.-T. Tu S.-H. Yang P.-S. Hsu S.-P. Lee W.-H. Ho C.-T. (2016). Apple polyphenol phloretin inhibits colorectal cancer cell growth via inhibition of the type 2 glucose transporter and activation of p53-mediated signaling. J. Agric. Food Chem. 64, 6826–6837. 10.1021/acs.jafc.6b02861 27538679

[B106] Liu K. G. Kim J.-I. Olszewski K. Barsotti A. M. Morris K. Lamarque C. (2020). Discovery and optimization of glucose uptake inhibitors. J. Med. Chem. 63, 5201–5211. 10.1021/acs.jmedchem.9b02153 32282207

[B107] Liu R. Wang X. Shen Y. He A. (2021). Long non-coding RNA-based glycolysis-targeted cancer therapy: feasibility, progression and limitations. Mol. Biol. Rep. 48, 2713–2727. 10.1007/s11033-021-06247-7 33704659

[B108] Liu W. Kang L. Han J. Wang Y. Shen C. Yan Z. (2018). miR-342-3p suppresses hepatocellular carcinoma proliferation through inhibition of IGF-1R-mediated Warburg effect. Onco. Targets. Ther. 11, 1643–1653. 10.2147/OTT.S161586 29615839 PMC5870664

[B109] Liu Y. Cao Y. Zhang W. Bergmeier S. Qian Y. Akbar H. (2012). A small-molecule inhibitor of glucose transporter 1 downregulates glycolysis, induces cell-cycle arrest, and inhibits cancer cell growth *in vitro* and *in vivo* . Mol. Cancer Ther. 11, 1672–1682. 10.1158/1535-7163.MCT-12-0131 22689530

[B110] Lloyd K. P. Ojelabi O. A. De Zutter J. K. Carruthers A. (2017). Reconciling contradictory findings: Glucose transporter 1 (GLUT1) functions as an oligomer of allosteric, alternating access transporters. J. Biol. Chem. 292, 21035–21046. 10.1074/jbc.M117.815589 29066623 PMC5743077

[B111] Lopez-Lazaro M. (2008). The Warburg effect: Why and how do cancer cells activate glycolysis in the presence of oxygen? Anticancer. Agents Med. Chem. 8, 305–312. 10.2174/187152008783961932 18393789

[B112] Lu H. Buchan R. J. Cook S. A. (2010). MicroRNA-223 regulates Glut4 expression and cardiomyocyte glucose metabolism. Cardiovasc. Res. 86, 410–420. 10.1093/cvr/cvq010 20080987

[B113] Lunt S. Y. Vander Heiden M. G. (2011). Aerobic glycolysis: meeting the metabolic requirements of cell proliferation. Annu. Rev. Cell Dev. Biol. 27, 441–464. 10.1146/annurev-cellbio-092910-154237 21985671

[B114] Macheda M. L. Rogers S. Best J. D. (2005). Molecular and cellular regulation of glucose transporter (GLUT) proteins in cancer. J. Cell. Physiol. 202, 654–662. 10.1002/jcp.20166 15389572

[B115] Maria Z. Campolo A. R. Lacombe V. A. (2015). Diabetes alters the expression and translocation of the insulin-sensitive glucose transporters 4 and 8 in the atria. PLoS ONE 10, e0146033. 10.1371/journal.pone.0146033 26720696 PMC4697822

[B116] Martell R. L. Slapak C. A. Levy S. B. (1997). Effect of glucose transport inhibitors on vincristine efflux in multidrug-resistant murine erythroleukaemia cells overexpressing the multidrug resistance-associated protein (MRP) and two glucose transport proteins, GLUT1 and GLUT3. Br. J. Cancer 75, 161–168. 10.1038/bjc.1997.27 9010020 PMC2063264

[B117] Masoud G. N. Li W. (2015). HIF-1α pathway: role, regulation and intervention for cancer therapy. Acta Pharm. Sin. B 5, 378–389. 10.1016/j.apsb.2015.05.007 26579469 PMC4629436

[B118] Mattmiller S. A. Corl C. M. Gandy J. C. Loor J. J. Sordillo L. M. (2011). Glucose transporter and hypoxia-associated gene expression in the mammary gland of transition dairy cattle. J. Dairy Sci. 94, 2912–2922. 10.3168/jds.2010-3936 21605761

[B119] McBrayer S. K. Cheng J. C. Singhal S. Krett N. L. Rosen S. T. Shanmugam M. (2012). Multiple myeloma exhibits novel dependence on GLUT4, GLUT8, and GLUT11: implications for glucose transporter-directed therapy. Blood 119, 4686–4697. 10.1182/blood-2011-09-377846 22452979 PMC3367873

[B120] Medina R. A. Meneses A. M. Vera J. C. Guzman C. Nualart F. Astuya A. (2003). Estrogen and progesterone up-regulate glucose transporter expression in ZR-75-1 human breast cancer cells. Endocrinology 144, 4527–4535. 10.1210/en.2003-0294 12960090

[B121] Medina R. A. Meneses A. M. Vera J. C. Gúzman C. Nualart F. Rodriguez F. (2004). Differential regulation of glucose transporter expression by estrogen and progesterone in Ishikawa endometrial cancer cells. J. Endocrinol. 182, 467–478. 10.1677/joe.0.1820467 15350188

[B122] Medina R. A. Owen G. I. (2002). Glucose transporters: expression, regulation and cancer. Biol. Res. 35, 9–26. 10.4067/s0716-97602002000100004 12125211

[B123] Melstrom L. G. Salabat M. R. Ding X.-Z. Milam B. M. Strouch M. Pelling J. C. (2008). Apigenin inhibits the GLUT-1 glucose transporter and the phosphoinositide 3-kinase/Akt pathway in human pancreatic cancer cells. Pancreas 37, 426–431. 10.1097/MPA.0b013e3181735ccb 18953257

[B124] Memariani Z. Abbas S. Q. ul Hassan S. S. Ahmadi A. Chabra A. (2021). Naringin and naringenin as anticancer agents and adjuvants in cancer combination therapy: Efficacy and molecular mechanisms of action, a comprehensive narrative review. Pharmacol. Res. 171, 105264. 10.1016/j.phrs.2020.105264 33166734

[B125] Meneses A. M. Medina R. A. Kato S. Pinto M. Jaque M. P. Lizama I. (2008). Regulation of GLUT3 and glucose uptake by the cAMP signalling pathway in the breast cancer cell line ZR-75. J. Cell. Physiol. 214, 110–116. 10.1002/jcp.21166 17559076

[B126] Mitsuhashi K. Senmaru T. Fukuda T. Yamazaki M. Shinomiya K. Ueno M. (2016). Testosterone stimulates glucose uptake and GLUT4 translocation through LKB1/AMPK signaling in 3T3-L1 adipocytes. Endocrine 51, 174–184. 10.1007/s12020-015-0666-y 26100787

[B127] Moldogazieva N. T. Mokhosoev I. M. Terentiev A. A. (2020). Metabolic heterogeneity of cancer cells: An interplay between HIF-1, GLUTs, and AMPK. Cancers 12, 862. 10.3390/cancers12040862 PMC7226606 32252351

[B128] Monde N. (2018). “Glucose metabolism and carcinogenesis: The impact of the tumor suppressor p53,” in Ubanako njende ED1 - hafiz naveed shahzad (Rijeka: IntechOpen). 10.5772/intechopen.75976

[B129] Montané X. Kowalczyk O. Reig-Vano B. Bajek A. Roszkowski K. Tomczyk R. (2020). Current perspectives of the applications of polyphenols and flavonoids in cancer therapy. Molecules 25, 3342. 10.3390/molecules25153342 PMC7435624 32717865

[B130] Mori Y. Yamawaki K. Ishiguro T. Yoshihara K. Ueda H. Sato A. (2019). ALDH-dependent glycolytic activation mediates stemness and paclitaxel resistance in patient-derived spheroid models of uterine endometrial cancer. Stem Cell Rep. 13, 730–746. 10.1016/j.stemcr.2019.08.015 PMC6829754 31564647

[B131] Mueckler M. Caruso C. Baldwin S. A. Panico M. Blench I. Morris H. R. (1985). Sequence and structure of a human glucose transporter. Science 229, 941–945. 10.1126/science.3839598 3839598

[B132] Mueckler M. (1994). Facilitative glucose transporters. Eur. J. Biochem. 219, 713–725. 10.1111/j.1432-1033.1994.tb18550.x 8112322

[B133] Mueckler M. Makepeace C. (2009). Model of the exofacial substrate-binding site and helical folding of the human Glut1 glucose transporter based on scanning mutagenesis. Biochemistry 48, 5934–5942. 10.1021/bi900521n 19449892 PMC2776625

[B134] Mueckler M. Thorens B. (2013). The SLC2 (GLUT) family of membrane transporters. Mol. Asp. Med. 34, 121–138. 10.1016/j.mam.2012.07.001 PMC4104978 23506862

[B135] Nagao A. Kobayashi M. Koyasu S. Chow C. C. T. Harada H. (2019). HIF-1-Dependent reprogramming of glucose metabolic pathway of cancer cells and its therapeutic significance. Int. J. Mol. Sci. 20, 238. 10.3390/ijms20020238 PMC6359724 30634433

[B136] Navale A. M. Paranjape A. N. (2016). Glucose transporters: physiological and pathological roles. Biophys. Rev. 8, 5–9. 10.1007/s12551-015-0186-2 PMC5425736 28510148

[B137] Nicholas D. A. Knight V. S. Tonsfeldt K. J. Terasaka T. Molinar-Inglis O. Stephens S. B. Z. (2020). GLUT1-mediated glycolysis supports GnRH-induced secretion of luteinizing hormone from female gonadotropes. Sci. Rep. 10, 13063. 10.1038/s41598-020-69913-z 32747664 PMC7400764

[B138] Niedzwiecki A. Roomi M. W. Kalinovsky T. Rath M. (2016). Anticancer efficacy of polyphenols and their combinations. Nutrients 8, 552. 10.3390/nu8090552 PMC5037537 27618095

[B139] Nomura N. Verdon G. Kang H. J. Shimamura T. Nomura Y. Sonoda Y. (2015). Structure and mechanism of the mammalian fructose transporter GLUT5. Nature 526, 397–401. 10.1038/nature14909 26416735 PMC4618315

[B140] Nualart F. Los Angeles García M. Medina R. A. Owen G. I. (2009). Glucose transporters in sex steroid hormone related cancer. Curr. Vasc. Pharmacol. 7, 534–548. 10.2174/157016109789043928 19485886

[B141] Obaid M. Udden S. M. N. Alluri P. Mandal S. S. (2021). LncRNA HOTAIR regulates glucose transporter Glut1 expression and glucose uptake in macrophages during inflammation. Sci. Rep. 11, 232. 10.1038/s41598-020-80291-4 33420270 PMC7794310

[B142] Ocaña M. C. Martínez-Poveda B. Marí-Beffa M. Quesada A. R. Medina M. Á. (2020). Fasentin diminishes endothelial cell proliferation, differentiation and invasion in a glucose metabolism-independent manner. Sci. Rep. 10, 6132. 10.1038/s41598-020-63232-z 32273578 PMC7145862

[B143] Oh S. Kim H. Nam K. Shin I. (2017). Glut1 promotes cell proliferation, migration and invasion by regulating epidermal growth factor receptor and integrin signaling in triple-negative breast cancer cells. BMB Rep. 50, 132–137. 10.5483/bmbrep.2017.50.3.189 27931517 PMC5422025

[B144] Ojelabi O. DeZutter J. Lloyd K. Carruthers A. (2016). Novel small molecule, WZB117, competitively inhibit GLUT1-mediated glucose transport to halt cancer growth. FASEB J. 30, 1099. 10.1096/fasebj.30.1_supplement.1099.1

[B145] Ojelabi O. (2017). Small molecule modulation of GLUT1-mediated glucose transport. Morningside Grad. Sch. Biomed. Sci. Diss. Theses. 10.13028/M2R69F

[B146] Olszewski K. Barsotti A. Feng X.-J. Momcilovic M. Liu K. G. Kim J.-I. (2022). Inhibition of glucose transport synergizes with chemical or genetic disruption of mitochondrial metabolism and suppresses TCA cycle-deficient tumors. Cell Chem. Biol. 29, 423–435. 10.1016/j.chembiol.2021.10.007 34715056

[B147] Palorini R. Simonetto T. Cirulli C. Chiaradonna F. (2013). Mitochondrial complex I inhibitors and forced oxidative phosphorylation synergize in inducing cancer cell death. Int. J. Cell Biol. 2013, e243876. 10.1155/2013/243876 PMC3638674 23690779

[B148] Parker J. (2020). Glucose metabolism, energy production and regulation of cellular and whole-body metabolism. J. ACNEM 39 (1), 29–33.

[B149] Pedroza-Torres A. Romero-Córdoba S. L. Justo-Garrido M. Salido-Guadarrama I. Rodríguez-Bautista R. Montaño S. (2019). MicroRNAs in tumor cell metabolism: Roles and therapeutic opportunities. Front. Oncol. 9, 1404. 10.3389/fonc.2019.01404 31921661 PMC6917641

[B150] Pérez A. Ojeda P. Ojeda L. Salas M. Rivas C. I. Vera J. C. (2011). Hexose transporter GLUT1 harbors several distinct regulatory binding sites for flavones and tyrphostins. Biochemistry 50, 8834–8845. 10.1021/bi200748b 21899256

[B151] Phay J. E. Hussain H. B. Moley J. F. (2000). Cloning and expression analysis of a novel member of the facilitative glucose transporter family, SLC2A9 (GLUT9). Genomics 66, 217–220. 10.1006/geno.2000.6195 10860667

[B152] Phillips T. Ferraz I. Bell S. Clegg P. D. Carter S. D. Mobasheri A. (2005). Differential regulation of the GLUT1 and GLUT3 glucose transporters by growth factors and pro-inflammatory cytokines in equine articular chondrocytes. Vet. J. 169, 216–222. 10.1016/j.tvjl.2004.01.026 15727913

[B153] Pliszka M. Szablewski L. (2021). Glucose transporters as a target for anticancer therapy. Cancers 13, 4184. 10.3390/cancers13164184 34439338 PMC8394807

[B154] Potashnik R. Kozlovsky N. Ben-Ezra S. Rudich A. Bashan N. (1995). Regulation of glucose transport and GLUT-1 expression by iron chelators in muscle cells in culture. Am. J. Physiol. 269, E1052–E1058. 10.1152/ajpendo.1995.269.6.E1052 8572196

[B155] Pragallapati S. Manyam R. (2019). Glucose transporter 1 in health and disease. J. Oral Maxillofac. Pathol. 23, 443–449. 10.4103/jomfp.JOMFP_22_18 31942129 PMC6948067

[B156] Pyla R. Poulose N. Jun J. Y. Segar L. (2013). Expression of conventional and novel glucose transporters, GLUT1, -9, -10, and -12, in vascular smooth muscle cells. Am. J. Physiol. Cell Physiol. 304, C574–C589. 10.1152/ajpcell.00275.2012 23302780 PMC3671567

[B157] Raez L. E. Papadopoulos K. Ricart A. D. Chiorean E. G. DiPaola R. S. Stein M. N. (2013). A phase I dose-escalation trial of 2-deoxy-d-glucose alone or combined with docetaxel in patients with advanced solid tumors. Cancer Chemother. Pharmacol. 71, 523–530. 10.1007/s00280-012-2045-1 23228990

[B158] Rastogi S. Banerjee S. Chellappan S. Simon G. R. (2007). Glut-1 antibodies induce growth arrest and apoptosis in human cancer cell lines. Cancer Lett. 257, 244–251. 10.1016/j.canlet.2007.07.021 17910902

[B159] Reckzeh E. S. Karageorgis G. Schwalfenberg M. Ceballos J. Nowacki J. Stroet M. C. M. (2019). Inhibition of glucose transporters and glutaminase synergistically impairs tumor cell growth. Cell Chem. Biol. 26, 1214–1228. 10.1016/j.chembiol.2019.06.005 31303578

[B160] Reckzeh E. S. Waldmann H. (2020b). Development of glucose transporter (GLUT) inhibitors. Eur. J. Org. Chem. 2020, 2321–2329. 10.1002/ejoc.201901353 PMC7217229 32421048

[B161] Reckzeh E. S. Waldmann H. (2020a). Small-molecule inhibition of glucose transporters GLUT-1–4. ChemBioChem 21, 45–52. 10.1002/cbic.201900544 31553512 PMC7004114

[B162] Richter E. A. Hargreaves M. (2013). Exercise, GLUT4, and skeletal muscle glucose uptake. Physiol. Rev. 93, 993–1017. 10.1152/physrev.00038.2012 23899560

[B163] Roberts D. A. Wang L. Zhang W. Liu Y. Shriwas P. Qian Y. (2020). Isosteres of ester derived glucose uptake inhibitors. Bioorg. Med. Chem. Lett. 30, 127406. 10.1016/j.bmcl.2020.127406 32736210

[B164] Röckl K. S. C. Hirshman M. F. Brandauer J. Fujii N. Witters L. A. Goodyear L. J. (2007). Skeletal muscle adaptation to exercise training: AMP-activated protein kinase mediates muscle fiber type shift. Diabetes 56, 2062–2069. 10.2337/db07-0255 17513699

[B165] Roy S. Kumaravel S. Sharma A. Duran C. L. Bayless K. J. Chakraborty S. (2020). Hypoxic tumor microenvironment: Implications for cancer therapy. Exp. Biol. Med. 245, 1073–1086. 10.1177/1535370220934038 PMC7400722 32594767

[B166] Sadlecki P. Bodnar M. Grabiec M. Marszalek A. Walentowicz P. Sokup A. (2014). The role of hypoxia-inducible factor-1α, glucose transporter-1, (GLUT-1) and carbon anhydrase IX in endometrial cancer patients. Biomed. Res. Int. 2014, e616850. 10.1155/2014/616850 PMC3972900 24745019

[B167] Salehi B. Machin L. Monzote L. Sharifi-Rad J. Ezzat S. M. Salem M. A. (2020). Therapeutic potential of quercetin: New insights and perspectives for human health. ACS Omega 5, 11849–11872. 10.1021/acsomega.0c01818 32478277 PMC7254783

[B168] Samec M. Liskova A. Koklesova L. Samuel S. M. Zhai K. Buhrmann C. (2020). Flavonoids against the Warburg phenotype—concepts of predictive, preventive and personalised medicine to cut the gordian knot of cancer cell metabolism. EPMA J. 11, 377–398. 10.1007/s13167-020-00217-y 32843908 PMC7429635

[B169] Santos F. El-Dandachli A. Buggs-Saxton C. (2014). Regulation of glucose transporter 1 (Slc2a1) in the pituitary gonadotrope of mice after. Puberty 5, 1000138. 10.4172/2157-7536.1000138

[B170] Sawayama H. Ogata Y. Ishimoto T. Mima K. Hiyoshi Y. Iwatsuki M. (2019). Glucose transporter 1 regulates the proliferation and cisplatin sensitivity of esophageal cancer. Cancer Sci. 110, 1705–1714. 10.1111/cas.13995 30861255 PMC6500964

[B171] Schmidl S. Tamayo Rojas S. A. Iancu C. V. Choe J.-Y. Oreb M. (2021a). Molecular dynamics simulations of a chimeric androgen receptor protein (SPARKI) confirm the importance of the dimerization domain on DNA binding specificity. Front. Mol. Biosci. 7, 4. 10.3389/fmolb.2020.00004 PMC7005049 32083093

[B172] Schmidl S. Ursu O. Iancu C. V. Oreb M. Oprea T. I. Choe J. (2021b). Identification of new GLUT2-selective inhibitors through *in silico* ligand screening and validation in eukaryotic expression systems. Sci. Rep. 11, 13751. 10.1038/s41598-021-93063-5 34215797 PMC8253845

[B173] Schwartzenberg-Bar-Yoseph F. Armoni M. Karnieli E. (2004). The tumor suppressor p53 down-regulates glucose transporters GLUT1 and GLUT4 gene expression. Cancer Res. 64, 2627–2633. 10.1158/0008-5472.can-03-0846 15059920

[B174] Seleit I. Bakry O. A. Al-Sharaky D. R. Ragab R. A. A. Al-Shiemy S. A. (2017). Evaluation of hypoxia inducible factor-1α and glucose transporter-1 expression in non melanoma skin cancer: an immunohistochemical study. J. Clin. Diagn. Res. 11, EC09–EC16. 10.7860/JCDR/2017/25077.10022 PMC5535364 28764171

[B175] Semenza G. L. (2013). HIF-1 mediates metabolic responses to intratumoral hypoxia and oncogenic mutations. J. Clin. Invest. 123, 3664–3671. 10.1172/JCI67230 23999440 PMC3754249

[B176] Semenza G. L. (2010). HIF-1: upstream and downstream of cancer metabolism. Curr. Opin. Genet. Dev. 20, 51–56. 10.1016/j.gde.2009.10.009 19942427 PMC2822127

[B177] Senga S. S. Grose R. P. (2021). Hallmarks of cancer-the new testament. Open Biol. 11, 200358. 10.1098/rsob.200358 33465324 PMC7881179

[B178] Shiah S.-G. Chou S.-T. Chang J.-Y. (2021). MicroRNAs: Their role in metabolism, tumor microenvironment, and therapeutic implications in head and neck squamous cell carcinoma. Cancers 13, 5604. 10.3390/cancers13225604 34830755 PMC8615702

[B179] Shibuya K. Okada M. Suzuki S. Seino M. Seino S. Takeda H. (2014). Targeting the facilitative glucose transporter GLUT1 inhibits the self-renewal and tumor-initiating capacity of cancer stem cells. Oncotarget 6, 651–661. 10.18632/oncotarget.2892 PMC4359246 25528771

[B180] Shikhman A. R. Brinson D. C. Valbracht J. Lotz M. K. (2001). Cytokine regulation of facilitated glucose transport in human articular chondrocytes. J. Immunol. 167, 7001–7008. 10.4049/jimmunol.167.12.7001 11739520

[B181] Shim B. Y. Jung J. H. Lee K. M. Kim H. J. Hong S. H. Kim S. H. (2013). Glucose transporter 1 (GLUT1) of anaerobic glycolysis as predictive and prognostic values in neoadjuvant chemoradiotherapy and laparoscopic surgery for locally advanced rectal cancer. Int. J. Colorectal Dis. 28, 375–383. 10.1007/s00384-012-1542-3 22847606

[B182] Shin E. Koo J. S. (2021). Glucose metabolism and glucose transporters in breast cancer. Front. Cell Dev. Biol. 9, 728759. 10.3389/fcell.2021.728759 34552932 PMC8450384

[B183] Shriwas P. Chen X. Kinghorn A. D. Ren Y. (2020). Plant-derived glucose transport inhibitors with potential antitumor activity. Phytother. Res. 34, 1027–1040. 10.1002/ptr.6587 31823431 PMC7263379

[B184] Shriwas P. Qian Y. Wang X. Roberts D. Bergmeier S. Chen X. (2018). Abstract 2799: New-generation glucose transporter inhibitors targeting non-small cell lung cancer and triple-negative breast cancer. Cancer Res. 78, 2799. 10.1158/1538-7445.AM2018-2799 29599405

[B185] Shriwas P. Roberts D. Li Y. Wang L. Qian Y. Bergmeier S. (2021). A small-molecule pan-class I glucose transporter inhibitor reduces cancer cell proliferation *in vitro* and tumor growth *in vivo* by targeting glucose-based metabolism. Cancer Metab. 9, 14. 10.1186/s40170-021-00248-7 33771231 PMC8004435

[B186] Siebeneicher H. Bauser M. Buchmann B. Heisler I. Müller T. Neuhaus R. (2016a). Identification of novel GLUT inhibitors. Bioorg. Med. Chem. Lett. 26, 1732–1737. 10.1016/j.bmcl.2016.02.050 26949183

[B187] Siebeneicher H. Cleve A. Rehwinkel H. Neuhaus R. Heisler I. Müller T. (2016b). Identification and optimization of the first highly selective GLUT1 inhibitor BAY‐876. Chemmedchem 11, 2261–2271. 10.1002/cmdc.201600276 27552707 PMC5095872

[B188] Soni V. K. Mehta A. Ratre Y. K. Chandra V. Shukla D. Kumar A. (2021). Counteracting action of curcumin on high glucose-induced chemoresistance in hepatic carcinoma cells. Front. Oncol. 11, 738961. 10.3389/fonc.2021.738961 34692517 PMC8526934

[B189] Straus D. S. (2013). TNFα and IL-17 cooperatively stimulate glucose metabolism and growth factor production in human colorectal cancer cells. Mol. Cancer 12, 78. 10.1186/1476-4598-12-78 23866118 PMC3725176

[B190] Stuart C. A. Howell M. E. A. Zhang Y. Yin D. (2009). Insulin-stimulated translocation of glucose transporter (GLUT) 12 parallels that of GLUT4 in normal muscle. J. Clin. Endocrinol. Metab. 94, 3535–3542. 10.1210/jc.2009-0162 19549745 PMC2741719

[B191] Suwabe Y. Nakano R. Namba S. Yachiku N. Kuji M. Sugimura M. (2021). Involvement of GLUT1 and GLUT3 in the growth of canine melanoma cells. PLoS ONE 16, e0243859. 10.1371/journal.pone.0243859 33539362 PMC7861381

[B192] Szablewski L. (2013). Expression of glucose transporters in cancers. Biochim. Biophys. Acta 1835, 164–169. 10.1016/j.bbcan.2012.12.004 23266512

[B193] Szablewski L. (2022). Glucose transporters as markers of diagnosis and prognosis in cancer diseases. Oncol. Rev. 16, 561. 10.4081/oncol.2022.561 35340885 PMC8941341

[B194] Takaguri A. Inoue S. Kubo T. Satoh K. (2016). AMPK activation by prolonged stimulation with interleukin-1β contributes to the promotion of GLUT4 translocation in skeletal muscle cells. Cell Biol. Int. 40, 1204–1211. 10.1002/cbin.10673 27569904

[B195] Tan Q. Huang Q. Ma Y. L. Mao K. Yang G. Luo P. (2018). Potential roles of IL-1 subfamily members in glycolysis in disease. Cytokine Growth Factor Rev. 44, 18–27. 10.1016/j.cytogfr.2018.11.001 30470512

[B196] Temre M. K. Yadav S. Goel Y. Pandey S. K. Kumar A. Singh S. M. (2022). Glutor, a glucose transporter inhibitor, exerts antineoplastic action on tumor cells of thymic origin: Implication of modulated metabolism, survival, oxidative stress, mitochondrial membrane potential, pH homeostasis, and chemosensitivity. Front. Oncol. 12, 925666. 10.3389/fonc.2022.925666 35847943 PMC9279700

[B197] Thorens B. Mueckler M. (2010). Glucose transporters in the 21st century. Am. J. Physiol. Endocrinol. Metab. 298, E141–E145. 10.1152/ajpendo.00712.2009 20009031 PMC2822486

[B198] Tilekar K. Upadhyay N. Iancu C. V. Pokrovsky V. Choe J. Ramaa C. S. (2020). Power of two: combination of therapeutic approaches involving glucose transporter (GLUT) inhibitors to combat cancer. Biochim. Biophys. Acta. Rev. Cancer 1874, 188457. 10.1016/j.bbcan.2020.188457 33096154 PMC7704680

[B199] Torrance C. J. Devente J. E. Jones J. P. Dohm G. L. (1997). Effects of thyroid hormone on GLUT4 glucose transporter gene expression and NIDDM in rats. Endocrinology 138, 1204–1214. 10.1210/endo.138.3.4981 9048628

[B200] Trendowski M. (2015). Using cytochalasins to improve current chemotherapeutic approaches. Anticancer. Agents Med. Chem. 15, 327–335. 10.2174/1871520614666141016164335 25322987 PMC4485394

[B201] Tsakalozou E. Eckman A. M. Bae Y. (2012). Combination effects of docetaxel and doxorubicin in hormone-refractory prostate cancer cells. Biochem. Res. Int. 2012, e832059. 10.1155/2012/832059 PMC3395329 22811914

[B202] Tuccinardi T. Granchi C. Iegre J. Paterni I. Bertini S. Macchia M. (2013). Oxime-based inhibitors of glucose transporter 1 displaying antiproliferative effects in cancer cells. Bioorg. Med. Chem. Lett. 23, 6923–6927. 10.1016/j.bmcl.2013.09.037 24200808

[B203] Uldry M. Ibberson M. Hosokawa M. Thorens B. (2002). GLUT2 is a high affinity glucosamine transporter. FEBS Lett. 524, 199–203. 10.1016/s0014-5793(02)03058-2 12135767

[B204] Uldry M. Thorens B. (2004). The SLC2 family of facilitated hexose and polyol transporters. Pflugers Arch. 447, 480–489. 10.1007/s00424-003-1085-0 12750891

[B205] Ung P. M.-U. Song W. Cheng L. Zhao X. Hu H. Chen L. (2016). Inhibitor discovery for the human GLUT1 from homology modeling and virtual screening. ACS Chem. Biol. 11, 1908–1916. 10.1021/acschembio.6b00304 27128978 PMC5356226

[B206] Vaupel P. Multhoff G. (2021). Revisiting the Warburg effect: historical dogma versus current understanding. J. Physiol. 599, 1745–1757. 10.1113/JP278810 33347611

[B207] Vera J. C. Reyes A. M. Cárcamo J. G. Velásquez F. V. Rivas C. I. Zhang R. H. (1996). Genistein is a natural inhibitor of hexose and dehydroascorbic acid transport through the glucose transporter, GLUT1. J. Biol. Chem. 271, 8719–8724. 10.1074/jbc.271.15.8719 8621505

[B208] Vick H. Diedrich D. F. Baumann K. (1973). Reevaluation of renal tubular glucose transport inhibition by phlorizin analogs. Am. J. Physiol. 224, 552–557. 10.1152/ajplegacy.1973.224.3.552 4691268

[B209] Wang N. Zhang S. Yuan Y. Xu H. Defossa E. Matter H. (2022). Molecular basis for inhibiting human glucose transporters by exofacial inhibitors. Nat. Commun. 13, 2632. 10.1038/s41467-022-30326-3 35552392 PMC9098912

[B210] Wang W.-D. Zhu J.-L. Zhou S.-H. Fan J. Bao Y.-Y. (2020). shRNA Glut-1 inhibits cell viability, apoptosis and migration of laryngeal carcinoma HEp-2 cells through regulating Beclin-1-mediated autophagy. bioRxiv. 10.1101/2020.02.24.962449

[B211] Wang Y.-D. Li S.-J. Liao J.-X. (2013). Inhibition of glucose transporter 1 (GLUT1) chemosensitized head and neck cancer cells to cisplatin. Technol. Cancer Res. Treat. 12, 525–535. 10.7785/tcrt.2012.500343 23617290

[B212] Wang Y. Jiang Y. Chen L. (2020). Role of miR-218-GREM1 axis in epithelial-mesenchymal transition of oral squamous cell carcinoma: An *in vivo* and vitro study based on microarray data. J. Cell. Mol. Med. 24, 13824–13836. 10.1111/jcmm.15972 33107676 PMC7754042

[B213] Warburg O. (1956). On the origin of cancer cells. Science 123, 309–314. 10.1126/science.123.3191.309 13298683

[B214] Warburg O. Wind F. Negelein E. (1927). The metabolism of tumors in the body. J. Gen. Physiol. 8, 519–530. 10.1085/jgp.8.6.519 19872213 PMC2140820

[B215] Weng H.-C. Sung C.-J. Hsu J.-L. Leu W.-J. Guh J.-H. Kung F.-L. (2022). The combination of a novel GLUT1 inhibitor and cisplatin synergistically inhibits breast cancer cell growth by enhancing the DNA damaging effect and modulating the akt/mTOR and MAPK signaling pathways. Front. Pharmacol. 13, 879748. 10.3389/fphar.2022.879748 35662690 PMC9160228

[B216] White M. A. Tsouko E. Lin C. Rajapakshe K. Spencer J. M. Wilkenfeld S. R. (2018). GLUT12 promotes prostate cancer cell growth and is regulated by androgens and CaMKK2 signaling. Endocr. Relat. Cancer 25, 453–469. 10.1530/ERC-17-0051 29431615 PMC5831527

[B217] Widdas W. F. (1952). Inability of diffusion to account for placental glucose transfer in the sheep and consideration of the kinetics of a possible carrier transfer. J. Physiol. 118, 23–39. 10.1113/jphysiol.1952.sp004770 13000688 PMC1392425

[B218] Wieman H. L. Wofford J. A. Rathmell J. C. (2007). Cytokine stimulation promotes glucose uptake via phosphatidylinositol-3 Kinase/akt regulation of Glut1 activity and trafficking. Mol. Biol. Cell 18, 1437–1446. 10.1091/mbc.E06-07-0593 17301289 PMC1838986

[B219] Wilson C. Contreras-Ferrat A. Venegas N. Osorio-Fuentealba C. Pávez M. Montoya K. (2013). Testosterone increases GLUT4-dependent glucose uptake in cardiomyocytes. J. Cell. Physiol. 228, 2399–2407. 10.1002/jcp.24413 23757167

[B221] Wood T. E. Dalili S. Simpson C. D. Hurren R. Mao X. Saiz F. S. (2008). A novel inhibitor of glucose uptake sensitizes cells to FAS-induced cell death. Mol. Cancer Ther. 7, 3546–3555. 10.1158/1535-7163.MCT-08-0569 19001437

[B222] Wright E. M. Loo D. D. F. Hirayama B. A. (2011). Biology of human sodium glucose transporters. Physiol. Rev. 91, 733–794. 10.1152/physrev.00055.2009 21527736

[B223] Wright E. M. (2020). SGLT2 and cancer. Pflugers Arch. 472, 1407–1414. 10.1007/s00424-020-02448-4 32820343 PMC7462911

[B224] Wu K.-H. Ho C.-T. Chen Z.-F. Chen L.-C. Whang-Peng J. Lin T.-N. (2018). The apple polyphenol phloretin inhibits breast cancer cell migration and proliferation via inhibition of signals by type 2 glucose transporter. J. Food Drug Anal. 26, 221–231. 10.1016/j.jfda.2017.03.009 29389559 PMC9332637

[B225] Wu Q. ba-alawi W. Deblois G. Cruickshank J. Duan S. Lima-Fernandes E. (2020). GLUT1 inhibition blocks growth of RB1-positive triple negative breast cancer. Nat. Commun. 11, 4205. 10.1038/s41467-020-18020-8 32826891 PMC7442809

[B226] Xintaropoulou C. Ward C. Wise A. Marston H. Turnbull A. Langdon S. P. (2015). A comparative analysis of inhibitors of the glycolysis pathway in breast and ovarian cancer cell line models. Oncotarget 6, 25677–25695. 10.18632/oncotarget.4499 26259240 PMC4694858

[B227] Xu X.-J. Yuan J. Sun W.-J. Chen Q.-Y. Lin Y. Tang L. (2018). Inhibition of microRNA-218 promotes oral squamous cell carcinoma growth by targeting GLUT1 to affect glucose metabolism. Eur. Rev. Med. Pharmacol. Sci. 22, 7726–7734. 10.26355/eurrev_201811_16394 30536316

[B228] Yamamoto T. Seino Y. Fukumoto H. Koh G. Yano H. Inagaki N. (1990). Over-expression of facilitative glucose transporter genes in human cancer. Biochem. Biophys. Res. Commun. 170, 223–230. 10.1016/0006-291X(90)91263-R 2372287

[B229] Yamasaki T. Seki N. Yoshino H. Itesako T. Yamada Y. Tatarano S. (2013). Tumor-suppressive microRNA-1291 directly regulates glucose transporter 1 in renal cell carcinoma. Cancer Sci. 104, 1411–1419. 10.1111/cas.12240 23889809 PMC7654250

[B230] Yan Q. Lu Y. Zhou L. Chen J. Xu H. Cai M. (2018). Mechanistic insights into GLUT1 activation and clustering revealed by super-resolution imaging. Proc. Natl. Acad. Sci. U. S. A. 115, 7033–7038. 10.1073/pnas.1803859115 29915035 PMC6142262

[B231] Yan S.-X. Luo X.-M. Zhou S.-H. Bao Y.-Y. Fan J. Lu Z.-J. (2013). Effect of antisense oligodeoxynucleotides glucose transporter-1 on enhancement of radiosensitivity of laryngeal carcinoma. Int. J. Med. Sci. 10, 1375–1386. 10.7150/ijms.6855 23983599 PMC3753417

[B232] Yang C. Jiang L. Zhang H. Shimoda L. A. DeBerardinis R. J. Semenza G. L. (2014). Analysis of hypoxia-induced metabolic reprogramming. Methods Enzymol. 542, 425–455. 10.1016/B978-0-12-416618-9.00022-4 24862279

[B233] Yang H. Zhang M.-Z. Sun H. Chai Y. Li X. Jiang Q. (2021). A novel microcrystalline BAY-876 formulation achieves long-acting antitumor activity against aerobic glycolysis and proliferation of hepatocellular carcinoma. Front. Oncol. 11, 783194. 10.3389/fonc.2021.783194 34869036 PMC8636331

[B234] Yang H. Zhong J.-T. Zhou S.-H. Han H.-M. (2019). Roles of GLUT-1 and HK-II expression in the biological behavior of head and neck cancer. Oncotarget 10, 3066–3083. 10.18632/oncotarget.24684 31105886 PMC6508962

[B235] Yao Z. Xie F. Li M. Liang Z. Xu W. Yang J. (2017). Oridonin induces autophagy via inhibition of glucose metabolism in p53-mutated colorectal cancer cells. Cell Death Dis. 8, e2633. 10.1038/cddis.2017.35 28230866 PMC5386482

[B236] Zambrano A. Molt M. Uribe E. Salas M. (2019). Glut 1 in cancer cells and the inhibitory action of resveratrol as A potential therapeutic strategy. Int. J. Mol. Sci. 20, 3374. 10.3390/ijms20133374 PMC6651361 31324056

[B237] Zha X. Hu Z. Ji S. Jin F. Jiang K. Li C. (2015). NFκB up-regulation of glucose transporter 3 is essential for hyperactive mammalian target of rapamycin-induced aerobic glycolysis and tumor growth. Cancer Lett. 359, 97–106. 10.1016/j.canlet.2015.01.001 25578782

[B238] Zhan T. Digel M. Küch E.-M. Stremmel W. Füllekrug J. (2011). Silybin and dehydrosilybin decrease glucose uptake by inhibiting GLUT proteins. J. Cell. Biochem. 112, 849–859. 10.1002/jcb.22984 21328458

[B239] Zhao F. Ming J. Zhou Y. Fan L. (2016). Inhibition of Glut1 by WZB117 sensitizes radioresistant breast cancer cells to irradiation. Cancer Chemother. Pharmacol. 77, 963–972. 10.1007/s00280-016-3007-9 27011212

[B241] Zhao M. Zhang Z. (2016). Glucose transporter regulation in cancer: A profile and the loops. Crit. Rev. Eukaryot. Gene Expr. 26, 223–238. 10.1615/CritRevEukaryotGeneExpr.2016016531 27650986

[B242] Zhao T. Zhu Y. Morinibu A. Kobayashi M. Shinomiya K. Itasaka S. (2014). HIF-1-mediated metabolic reprogramming reduces ROS levels and facilitates the metastatic colonization of cancers in lungs. Sci. Rep. 4, 3793. 10.1038/srep03793 24452734 PMC3899644

[B243] Zhao X. Lu C. Chu W. Zhang B. Zhen Q. Wang R. (2017). MicroRNA-124 suppresses proliferation and glycolysis in non-small cell lung cancer cells by targeting AKT-GLUT1/HKII. Tumour Biol. 39, 1010428317706215. 10.1177/1010428317706215 28488541

[B244] Zhou X. Fenical W. (2016). The unique chemistry and biology of the piericidins. J. Antibiot. (Tokyo) 69, 582–593. 10.1038/ja.2016.71 27301663

[B245] Zhou Z. Ibekwe E. Chornenkyy Y. (2018). Metabolic alterations in cancer cells and the emerging role of oncometabolites as drivers of neoplastic change. Antioxidants 7, 16. 10.3390/antiox7010016 PMC5789326 29342092

